# Application of biodegradable implants in pediatric orthopedics: shifting from absorbable polymers to biodegradable metals

**DOI:** 10.1016/j.bioactmat.2025.04.001

**Published:** 2025-04-10

**Authors:** Yunan Lu, Ting Zhang, Kai Chen, Federico Canavese, Chenyang Huang, Hongtao Yang, Jiahui Shi, Wubing He, Yufeng Zheng, Shunyou Chen

**Affiliations:** a Department of Pediatric Orthopedics, Fuzhou Second General Hospital, The Third Clinical Medicine College of Fujian Medical University, 47th Shangteng Road of Cangshan District, Fuzhou, 350007, Fujian, China; b School of Materials Science and Engineering, Peking University, Beijing, 100871, China; c Department of Emergency Trauma Surgery, Shengli Clinical Medical College of Fujian Medical University, Shengli Hospital affiliated to Fuzhou University, Fuzhou, 350001, China; d Orthopedic and Traumatology Department, IRCCS Istituto Giannina Gaslini, DISC-Dipartimento di scienze chirurgiche e diagnostiche integrate, University of Genova, Genova, Italy; e School of Engineering Medicine, School of Biological Science and Medical Engineering, Beihang University, Beijing, 100191, China; f Shenzhen Key Laboratory of Spine Surgery, Department of Spine Surgery, Peking University Shenzhen Hospital, Shenzhen Peking University-The Hong Kong University of Science and Technology Medical Center, Shenzhen, 518036, Guangdong, China; g Fujian Provincial Clinical Medical Research Center for First Aid and Rehabilitation in Orthopedic Trauma (2020Y2014), Fuzhou, 350007, China; h Key Clinical Specialty of Fujian Province and Fuzhou City (20220104), Fuzhou, China

**Keywords:** Pediatric orthopedics, Percutaneous internal fixation, Orthopedic implant, Absorbable polymers, Biodegradable metals

## Abstract

Over the past two decades, advances in pediatric orthopedics and closed reduction combined with percutaneous internal fixation techniques have led to significant growth in pediatric orthopedics surgery. Implants such as Kirschner-wires, cannulated screws and elastic stabilization intramedullary nails are commonly used in these procedures. However, traditional implants made of metal or inert materials are not absorbable, leading to complications that affect treatment outcomes. To address this issue, absorbable materials with excellent mechanical properties, good biocompatibility, and controlled degradation rates have been developed and applied in clinical practice. These materials include absorbable polymers and biodegradable metals. This article provides a comprehensive summary of these resorbable materials from a clinician's perspective. In addition, an in-depth discussion of the feasibility of their clinical applications and related research in pediatric orthopedics is included. We found that the applications of absorbable implants in pediatric orthopedics are shifting from absorbable polymers to biodegradable metals and emphasize that the functional characteristics of resorbable materials must be coordinated and complementary to the treatment in pediatric orthopedics.

## Highlights

• The characteristics of the pediatric skeleton and resorbable implants are discussed.
• The applications of resorbable implants in pediatric orthopaedics are summarized.
• Resorbable materials must be coordinated with the treatment in pediatric orthopedics.

## Graphical abstract

**Figure:**
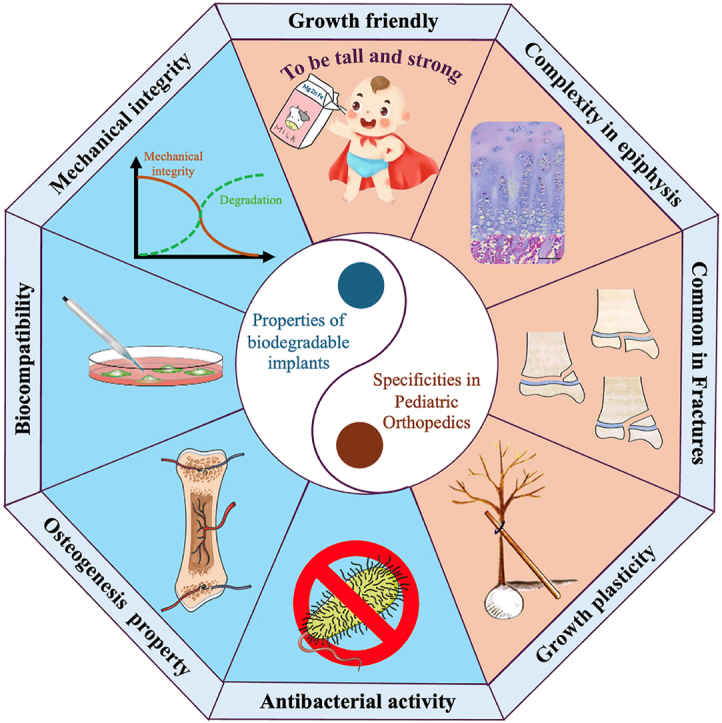

## 1 Introduction

Biodegradable materials have gained widespread application in clinical fields such as orthopedics, oral surgery, cardiovascular interventions and surgical sutures. While existing orthopedic reviews extensively cover their use in trauma, spinal and sports medicine, pediatric orthopedics - a subspecialty often underscored by the axiom “children are not little adults” - remains underrepresented in the literature [[1], [2], [3]]. The unique skeletal developmental patterns of children, including dynamic growth plates and different material tolerances, require specialized internal fixation strategies that differ from adult protocols. Implants such as Kirschner wires (K-wires), cannulated screws (C-screws), and elastic stable intramedullary nails (ESIN) are commonly used in children and are made of metals such as stainless steel and Titanium (Ti) or Cobalt-Chromium (Co-Cr) alloy. While metal implants provide effective and stable mechanical support, they also have some potential disadvantages when used in pediatric patients: 1) are not biodegradable and may irritate soft tissues, causing pain and discomfort if left in the body for prolonged periods; 2) may cause skin irritation, ulceration, or infection at the tip of the K-wires or ESIN when it protrudes from the bone to facilitate removal; 3) may create a tethering effect on the growth plate or cause physeal bars that ultimately impair bone growth; 4) may interfere with CT or MRI scans; 5) may require a second surgical procedure for removal, which can be particularly difficult, sometimes impossible, if left in place for long periods of time [4,5].

In recent years, advances in materials science have highlighted the advantages of bioabsorbable materials, and the literature on their use in orthopedic surgery has grown steadily [6]. The FDA has approved biostable polymers such as ultra-high molecular weight polyethylene (UHMWPE), polyurethane (PU), polymethyl methacrylate (PMMA), and polyetheretherketone (PEEK) as orthopedic implants [7]. These materials offer excellent mechanical properties, good biocompatibility, non-cytotoxicity, low revision rates, are physeal growth friendly, do not require removal, and do not interfere with imaging [8,9]. However, residual toxic small molecules or wear products can still have unpredictable effects on tissue healing [10].

The main purpose of this paper is to review the existing literature on resorbable materials and summarize their clinical applications in pediatric orthopedic surgery.

## 2 Key issues in pediatric orthopedics

The growing skeleton has two peculiarities: the epiphyseal growth plate, which ensures longitudinal growth of the bones, and a specific fracture pattern. The latter is related to the structure of pediatric bone, which is more elastic due to its higher water content. Understanding these structures is critical to the effective use of degradable materials in pediatric orthopedics.

### 2.1 Characteristics of children's skeleton

The adult and pediatric skeletal systems share fundamental structural and functional similarities, but have critical differences in composition, volume, and anatomical regions. Both contain organic components, primarily collagen, and inorganic minerals like calcium and phosphorus, which confer flexibility and strength, respectively. Cortical (dense) and trabecular (spongy) bone tissues are present in both groups, fulfilling roles in support, organ protection, and hematopoiesis. However, distinctions arise in developmental dynamics. Children's bones possess epiphyseal plates (growth plates), cartilage-rich regions enabling longitudinal growth, which fuse by adulthood, halting height increase [11]. Bone composition varies: pediatric bones have higher collagen content, enhancing flexibility and reducing brittleness, whereas adult bones are more mineralized, prioritizing strength over pliability [12]. This explains why children often sustain greenstick fractures (incomplete breaks) compared to complete fractures in adults. Bone volume and density also differ; children's skeletons undergo rapid modeling, accumulating bone mass until peak density is achieved in early adulthood. Consequently, adults typically have greater bone mass but experience gradual decline with aging, increasing osteoporosis risk. Bone marrow composition also differs: pediatric bones contain more red marrow for blood cell production, while adults retain red marrow only in select sites (e.g., pelvis), transitioning much to yellow marrow for fat storage. Remodeling rates are higher in children due to growth demands and mechanical adaptation, whereas adult remodeling focuses on maintenance and repair [13].

In addition, the main difference between the bones of children and adults is the anatomical regions. The long bones of children can be identified in the four specific anatomical regions: the epiphysis, the physis, the metaphysis, and the diaphysis. In contrast, there are only two anatomical regions that can be identified in adult long bones: the metaphysis and the diaphysis [14]. The epiphyseal plate has four layers of chondrocytes: 1) the reserve zone (or reserve chondrocytes, RC), 2) the proliferative zone (or proliferative chondrocytes, PC), 3) the hypertrophic zone (or hypertrophic chondrocytes, HC), and 4) the calcification zone (or trabecular bone, TB) [15].

**Fig. 1:**
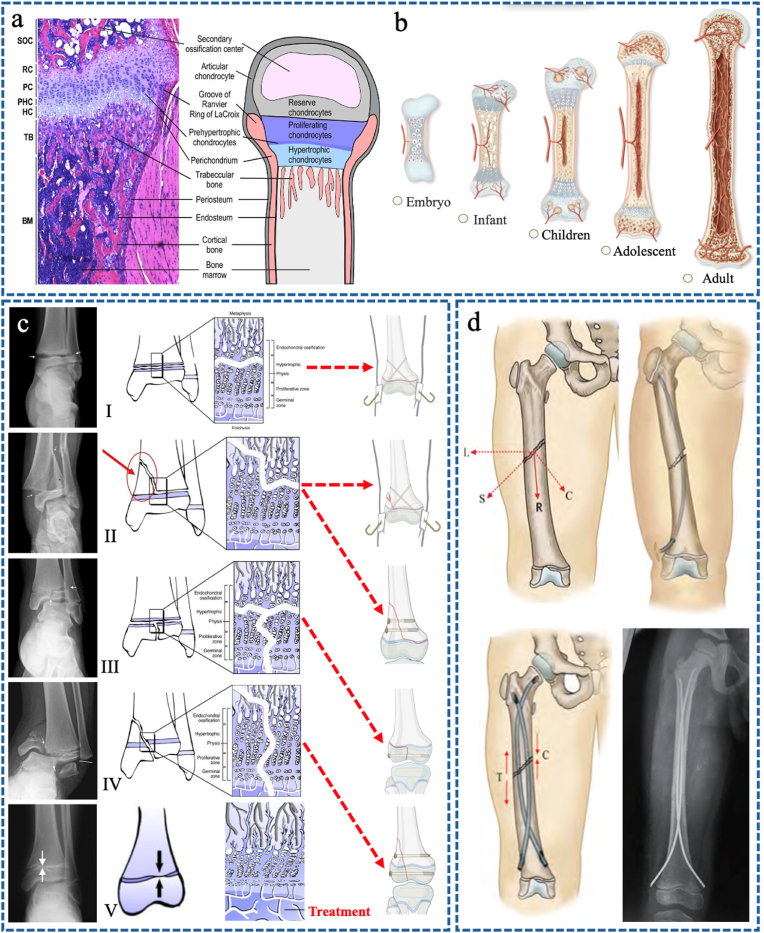
(a–b) Histological organization of the postnatal growth plate and an illustration of the gradual maturation of bones. Reproduced with permission from Ref. [15]. Copyright 2016, Wiley. (c) Classification of physeal fractures and corresponding treatment methods. Reproduced with permission from Ref. [20]. Copyright 2016, Springer. (d) Diaphyseal fracture and corresponding treatment of elastic stable intramedullary nails (ESIN). Reproduced with permission from Ref. [21]. Copyright 2021, Chinese Medical Association Publishing House.

The RC are located on the epiphyseal side and are grouped in clusters of two or three cells throughout the reserve zone, which generates new physeal chondrocytes [16]. The PCs are adjacent to the reserve zone and undergoe mitosis in columns parallel to the bone growth axis [17]. PCs mature and eventually increase their volume five to tenfold and evolve into HCs through a prehypertrophic state (prehypertrophic condrocytes, PHCs) [18]. The HCs then evolve into TB as matrix vesicles concentrate calcium and phosphate and enzymes such as alkaline phosphatase, which convert organic phosphates to inorganic phosphates, forming a longitudinal calcified septum around the terminal hypertrophic chondrocytes, restricting nutrient diffusion, leading to HC death and the formation of the calcified zone [19]. Capillaries and osteoblasts then penetrate the calcified zone and produce bone tissue. This creates the ossification zone, which gradually transforms into the metaphysis [14]. This process is called enchondral ossification and is specific to the growing skeleton (Fig. 1a–b).

Enchondral ossification occurs in the epiphyses with the appearance of secondary ossification centers (SOC). At birth, the epiphyses are cartilaginous (chondro-epiphyses) and complete their ossification over a period of several years, although not at the same time [22]. Once the epiphysis has ossified and fused with the metaphysis, the bone is mature and skeletal growth ceases [23]. The closing time of the epiphyseal growth plate is different in different sexes and different bones [24]; the first SOC is at the distal femoral epiphysis, while the last bone to complete ossification is the clavicle [25].

### 2.2 Fractures of the epiphyseal growth plate and principles of fixation

In 1963, Robert B. Salter and W. Robert Harris developed a prognostic classification system for epiphyseal fractures that identifies 5 types of lesions (Fig. 1c) [20,24,26].

In type I fractures, the fracture line passes through the TB zone of the epiphyseal growth plate, separating the epiphysis from the metaphysis. These fractures are more common in younger patients with a thicker physis, and because the RCs and PCs are not damaged, they have a good prognosis [27].

In type II fractures, the fracture line passes through the TB zone of the epiphyseal growth plate and extends into the metaphysis (Thurston-Holland fragment). These fractures also have a good prognosis because the RCs and PCs are not damaged [28].

Type III fractures are less common than Type I and II fractures, but carry a higher risk of early degenerative arthritis, intra-articular lesions, and epiphyseal growth plate damage because the fracture line originates in the TB but extends into the joint, damaging the hypertrophic, proliferative, and reserve zones [29].

Type IV fractures are also intra-articular like Type III fractures, but the fracture line extends from the metaphysis to the articular surface, crossing the epiphyseal growth plates and damaging all chondrocyte layers. Type IV fractures have a higher risk of premature physical closure, which may result in lower limb discrepancy or angular deformity [30].

Type V fractures result from compression of the entire epiphyseal growth plate [31]; this type of lesion can be seen in gymnasts with repetitive compression loading on an extended wrist [32].

Type I-IV epiphyseal growth plate injuries are fixed with a K-wire or C-screw, but not with plates, as metal hardware across the epiphyseal growth plate is not recommended because it may cause iatrogenic premature physical closure (Fig. 1c) [33]. In particular, C-screws may act as a tether, while K-wires should not cross the epiphyseal growth plate repeatedly or be larger than 6 % of the physeal cross-sectional area, as they may cause premature physeal closure and limit physeal growth [34].

### 2.3 Principles of fixation of diaphyseal and metaphyseal fractures

Another fracture pattern in children is diaphyseal fractures. These are often stabilized with ESIN, whose entry point does not damage the epiphyseal growth plate (Fig. 1d) [21].

**Fig. 2:**
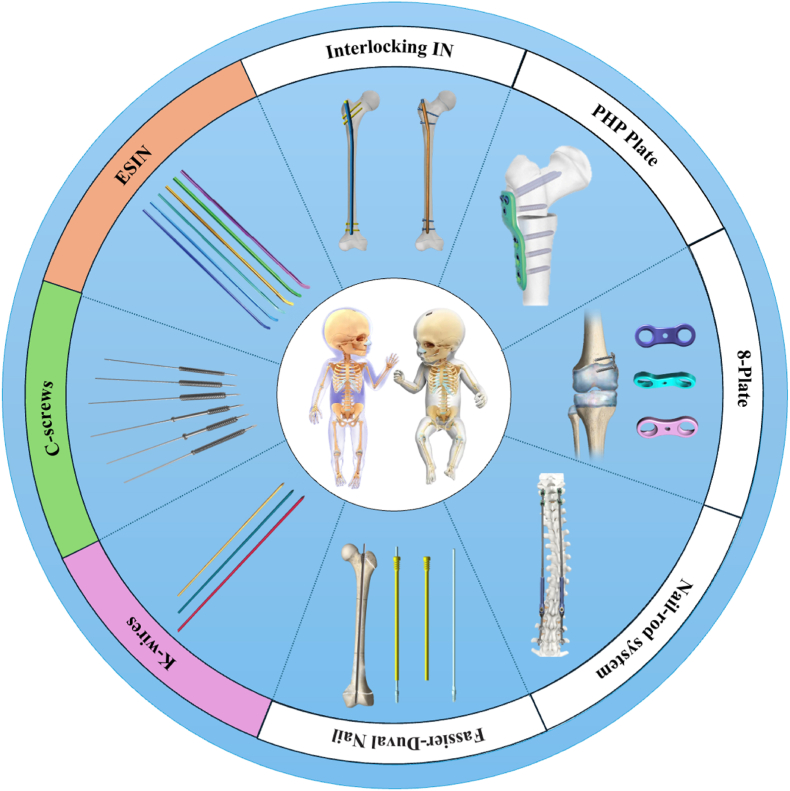
A common form of internal fixation in pediatric orthopedics.

The predominant fracture patterns in pediatric orthopedics include diaphyseal fractures of long bones and distal epiphyseal fractures. Commonly employed fixation modalities in skeletally immature patients include K-wires/pins, C-screws and ESIN. The main difference between C-screws and K-wires/pins is that the former have threads while the latter do not, while the main feature of ESIN is that its fixed pulp cavity often has some degree of elasticity. These are three of the most commonly used forms of internal fixation in pediatric orthopedics. They are three forms of the most commonly used internal fixation in pediatric orthopedics. Additional specialized devices such as interlocking intramedullary nails, Fassier-Duval nails, pediatric hip plates (PHP), guided growth 8-plates and spinal nail-rod systems are selectively utilized for complex injuries or growth-modulation procedures. Current clinical practice mainly relies on non-degradable titanium alloy implants for the latter several internal fixation methods, but biodegradable materials are mainly used for the first three (Fig. 2).

**Fig. 3:**
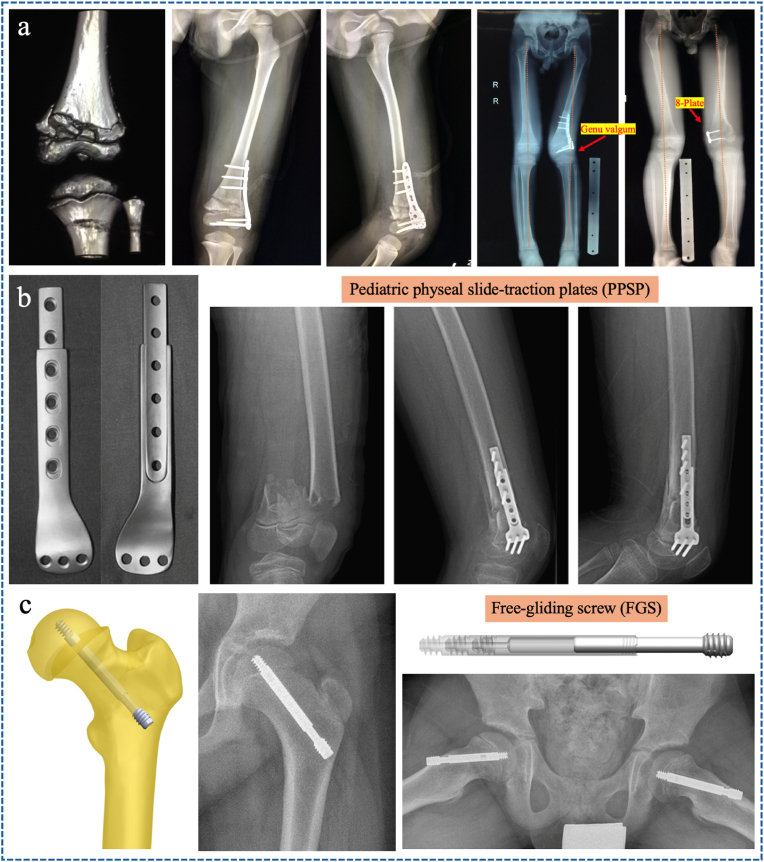
(a) An 8-year-old boy presented with a genu valgus after plate fixation of the left distal femoral fracture, after the lateral plate was removed, the deformity was corrected by temporary hemiepiphyseal arrest using an 8-Plate. (b) A 12-year-old boy underwent pediatric physeal slide-traction plate (PPSP) fixation for fracture of the left distal femur, 8 months aftersurgery seeing the extension of the plate without a limb-length inequality. Reproduced with permission from Ref. [37].Copyright 2012, Wilkins. (c) Free-gliding screw (FGS) for slipped capital femoral epiphysis.

On the other hand, open reduction and internal fixation (ORIF) with plates and screws is not well suited for pediatric long bones because the hardware may directly damage the periosteum, resulting in delayed union or non-union [35]. Similarly, plate and screw fixation of metaphyseal fractures is not recommended because it involves both the metaphyseal and epiphyseal bone (above and below the epiphyseal growth plate) and acts as an asymmetric tether, resulting in progressive angular deformity (Fig. 3a).

The use of rigid interlocking intramedullary nails is not recommended in children unless the nails have a lateral trochanteric entry point and the distal locking screws are above the epiphyseal growth plate [36].

### 2.4 Potential benefits of bioabsorbable implants in pediatric patients

The presence of the epiphyseal growth plate in the growing skeleton leads to a significant difference in the treatment concept between pediatric and adult traumatology, and misuse of treatment according to the adult concept or adult fixation pattern can lead to iatrogenic injuries in children.

Several internal fixation devices have been designed to preserve the epiphyseal growth plate, such as pediatric physeal slide-traction plates (PPSP) for distal femur (Fig. 3b) and proximal humerus fractures [37,38], extendable intramedullary rods (EIMR) for osteogenesis imperfecta or congenital tibial pseudarthrosis [39,40], free sliding screws (FSS) for slipped capital femoral epiphysis (Fig. 3c) [41,42], and new pediatric femoral neck system (NP-FNS) for pediatric femoral neck fractures [43].

In addition to sparing the epiphyseal growth plate, a second operation for implant removal is necessary. According to a questionnaire sent to 273 pediatric orthopedic surgeons and 99 non-pediatric specialists from around the world, more experienced surgeons recommended implant removal, especially for hip implants [44]. In addition, due to bone growth, inconsistencies between the incision made for implant placement and the incision required for implant removal are common, as the implant may have migrated during growth. The position of the implant and the difficulty of removal after cortical thickening are also possible in patients treated with ESIN [45,46]. Such problems can potentially be avoided with the use of resorbable (biodegradable) implants.

## 3 Overview of medical absorbable materials

Generally, the usage of absorbable or biodegradable implants in orthopedic surgeries is inevitable. The absorbable implant should fulfill their task and then disappear after finishing their tissue repair jobs [47]. In 1993, Speer et al. [48] first addressed the feasibility of bioabsorbable materials as orthopedic implants and described four basic concepts: 1) The bioabsorbable implant should have sufficient initial fixation strength to provide a stable connection between the soft tissue and the surrounding bone; 2) The bioabsorption profile of the implant should be well matched to the tissue regeneration period and provide favorable mechanical integrity; 3) The resorption of the bioresorbable implant should not be too slow or it will behave like its metal equivalent; 4) The composition of the bioresorbable implant should be completely safe. The kinetics degradation of biodegradable polymers and metals in pediatric versus adult patients may differ due to distinct physiological and biomechanical environments in developing long bones. In children, higher metabolic rates, dynamic bone remodeling, and differences in local pH/enzymatic activity could accelerate degradation kinetics compared to adults [49]. A review highlights that stochastic models accounting for age-dependent variability in degradation drivers - such as cellular activity, fluid flow dynamics, and mechanical loading patterns - are essential for predicting device performance across age groups [50]. While phenomenological models have elucidated general degradation trends for polymers in adults, pediatric-specific models must integrate developmental biology factors like growth plate dynamics, osteoclast/osteoblast activity and the biological activity of biomaterials ratios [49]. Table 1 lists the main characteristics of some typical bioabsorbable materials.

**Table 1:** Comparison between different kinds of biodegrdable orthopedic materials.

Absorbable Materials		Elastic modulus/GPa	Ultimate tensile strength (MPa)	Degrdation period	Degradation by-prodcuts	In vitro and in vivo performance and potential effects on bone formation	Ref.
Natural bone		3–20	130-180 (Compressive yiled strength)				[51]
Polymer materials	Polyglycolic acid (PGA)	>7.0	∼55	6–12 months	H_2_O, CO_2_	Relatively rapid degradation rate; Inflammatory reaction at the implantion sites	[52]
	Poly lactic acid (PLA)	2.8–4.0	∼50	Few years	H_2_O, CO_2_	Slow degradation rate; Poor biocompatibility; Hard to control degradation rate	[53,54]
	polylactic-co-glycolic acid (PLGA)	>7.0	∼50	More than 1–2 months	H_2_O, CO_2_	Good biocompatibility; Almost no inflammatory reaction; Poor osteointegration; Controblloable degradation rate	[55,56]
	Polycaprolactone (PCL)	0.2–0.3	∼10	2–4 years	H_2_O, CO_2_	Slow degradation rate; Poor mechanical strength; Poor load-bearing capacity	[57]
Biodegradable metals	Mg and Mg alloys	41–45	199–350	<1–1.5 years (MAGNEZIX)	Mg(OH)_2_, MgO, MgCl_2_, H_2_, MgCO_3_, Mg_3_(PO_4_)_2_	Necessary for bone growth; Prevention of skeletal fragility, osteoporosis, chronic chondrocalcinosis and myositis ossificans	[58,59]
	Zn and Zn alloys	94–110	167–520	>20 months	Zn(OH)_2_, ZnO, ZnCO_3_	Necessary for bone growth; Prevention of osteopenia and various skeletal abnormalities; Modulate bone turnover by stimulating osteoblast bone formation while inhibiting osteoclast differentiation; Increase bone strength	[60,61]
	Fe and Fe alloys	204–212	567–1059	>2 years	Fe(OH)_2_, Fe_3_O_4_	Necessary for bone growth	[62]

### 3.1 Polymer materials

Resorbable polymer materials are usually characterized by good mechanical strength, elasticity and plasticity [63]. In a physiological environment, resorbable polymers can be gradually degraded, with the degradation products being absorbed or excreted by the human body [64]. The rate of degradation can be controlled by the polymer structure. Currently, resorbable polymers can be of natural origin (cellulose, chitin, collagen) [65] or synthetic, and to date, polyglycolic acid (PGA), polylactic acid (PLA), polylactic-co-glycolic acid (PLGA), and polycaprolactone (PCL) [66] have been extensively studied and used in clinical practice.

#### 3.1.1 Polyglycolic acid (PGA)

Polyglycolic acid (PGA) is a polymer derived from α-monohydroxy acid (glycolic acid) during metabolism (molecular formular: (C_4_H_4_O_4_)_n_) [52]. In the physiological environment, the main degradation product of PGA is hydroxyacetic acid, which can be decomposed into CO_2_ or H_2_O and eliminated directly by urine [67]. However, the main drawbacks of PGA is its rapid degradation rate, and it has been reported that a mechanical strength integrity of ∼50 % and ∼10 % could be inspected for PGA scaffolds after 14 days or 28 days of degradation [68]. In a degradation period of 6–12 months, PGA can be totally degraded and absorbed by the body [69]. With its high elastic modulus, melting temperature, crystallinity, and predictable degradation rate, PGA was used to develop the first absorbable surgical suture in history, known as Dexon® [70], although its use in orthopedic surgery has been relatively limited due to its high degradation rate and loss of mechanical strength required for orthopedic implants [71,72]. In recent years, to effectively control the degradation rate of PGA scaffold, some methods, such as physical blending, chemical copolymerization with other materials and etc., have been adopted to modify PGA components.

#### 3.1.2 Polylactic acid (PLA)

Polylactic acid (PLA), also known as polylactide, is a new type of biodegradable material that is a polyester polymer obtained by polymerization of lactic acid as the main raw material (molecular formular: (C_3_H_4_O_2_)_n_). Made from starch derived from renewable plant resources such as corn, PLA is carbon neutral [73] and has superior mechanical properties and a controlled degradation rate compared to PGA. PLA sutures and rods were first developed in the 1960s and were considered for the treatment of mandibular fractures in dogs [74].

PLA degradation occurs in two stages. In the first stage, PLA is hydrolyzed to lactic acid and water-soluble oligomers. In the second stage, the lactic acid is converted to glycogen in the liver or enters the tricarboxylic acid cycle before being broken down to water and carbon dioxide and eliminated from the body [75]. In the natural environment, PLA exhibits a slow degradation rate, up to several years, and causes minimal inflammatory response in vivo [76].

The monomer of PLA is lactic acid, which has two optical isomers, resulting in three corresponding stereo configurations of PLA: levo polylactic acid (PLLA), dextro polylactic acid (PDLA), and racemic polylactic acid (PDLLA). Actually, lactic acid is a metabolic product produced by the human body during exercise and does not cause toxic effects to the human body under normal circumstances [77]. In addition, PLLA has a high modulus of elasticity (approximately 4.8 GPa), low elongation, and high tensile strength, suggesting it as a potential candidate for implants used at load-bearing sites. Some commercial PLLA implants, including MeniscalStinger®, Bio-Anchor®, BioScrew®, etc., have recently been introduced to the market [78]. In the clinical scenario, the degradation rate of PLLA is insufficient due to its high crystallinity, so the copolymerization of PLLA and PDLLA is often used to neutralize and adjust the actual performance by taking advantage of the high degradation rate and low crystallinity of PDLLA [79].

However, PLA has some disadvantages such as high brittleness and poor impact resistance. Therefore, to further enhance the strength and regulate the degradation rate of osteoimplants, self-reinforced materials or some novel composites have been developed to address these shortcomings. Recently, hydroxyapatite (HA), the major inorganic component of human and animal bone, has been blended with PLA to improve the mechanical strength, reduce the degradation rate, and provide durable mechanical integrity of implants [80,81]. In addition, the addition of HA can also improve the alkalinity balance, surface hydrophobicity, osteoinductivity, and protein adsorption capacity of the material.

#### 3.1.3 Polylactic-co-glycolic acid (PLGA)

Polylactic acid-co-glycolic acid (PLGA) is prepared by random polymerization of two monomers, lactic acid and glycolic acid [55]. It is a kind of functional and degradable organic compound, which has good biocompatibility, no toxicity, and good bagging and film-forming performance. It is widely used in pharmaceutical, medical engineering materials and modern industrial fields. In general, PGA exhibits a fast degradation rate, resulting in a rapid loss of mechanical strength. The properties of PLGA mainly depend on the copolymerization ratio, the molecular weight of the monomers and the copolymerization morphology. Previous studies have shown that the copolymerization ratio of LA/GA reaching up to 50/50 confers a faster degradation rate to the material over a period of 1–2 months [82]. PLGA usually shows good biocompatibility and does not cause any obvious inflammatory reactions [76]. As a result, PLGA is widely used as drug carriers, surgical sutures, and tissue engineering materials, while it is rarely used in orthopedics due to its low mechanical strength, high elasticity, and lack of osteogenic bioactivity [83,84].

#### 3.1.4 Polycaprolactone (PCL)

Polycaprolactone (PCL) is an organic polymer with the chemical formula (C_6_H_10_O_2_)n, which has the property of being well soluble in aromatic compounds, ketones and polar solvents. PCL has good biocompatibility, biodegradability, high crystallinity, low melting point and glass transition temperature [85]. It is easy to blend PCL with other polymers, and higher molecular weight usually means longer in vivo absorption time. In general, the in vivo degradation of PCL can be divided into two stages. First, the molecular weight of PCL will gradually decrease without deformation and mass reduction. In the second stage, there will be fragmentation and mass loss of the material, which will be continuously absorbed or excreted from the body [86]. To date, the PCL-based scaffolds have been widely investigated in bone tissue engineering [[87], [88], [89]]. For example, Yeo et al. recently successfully fabricated PCL-TCP scaffolds and the results showed that the scaffolds have the potential to degrade over a period of 5–6 months and have good mechanical properties [88].

#### 3.1.5 Biological mechanisms of absorbable polymers

Absorbable polymers, such as PLA, PGA, and their copolymers (PLGA), influence osteogenesis, angiogenesis, and immunomodulation through both direct and indirect mechanisms tied to their degradation kinetics and by-products. During hydrolysis, PLA and PLGA release lactic and glycolic acid, which transiently lower local pH, activating the PI3K/Akt signaling pathway in osteoblasts to enhance proliferation and differentiation via upregulation of Runx2 and osteocalcin [55]. Simultaneously, acidic microenvironments stimulate endothelial cells to secrete VEGF and FGF-2, promoting angiogenesis through ERK1/2 pathway activation [90]. However, excessive acidification may inhibit osteogenesis by suppressing alkaline phosphatase (ALP) activity [91]. In immunomodulation, PLGA degradation products polarize macrophages toward the M2 phenotype by inhibiting NF-κB-mediated pro-inflammatory cytokine release (e.g., TNF-α, IL-6) while upregulating anti-inflammatory IL-10 via STAT3 signaling [92]. Polycaprolactone (PCL), with slower degradation, minimizes acute inflammation but may delay tissue integration due to limited ion release [93]. Notably, polymer crystallinity and molecular weight further modulate these effects; for instance, low-crystallinity PLGA enhances macrophage phagocytosis and debris clearance, whereas high-crystallinity PLA persists longer, potentially triggering fibrous encapsulation [55]. Therefore, balancing degradation rates with bioactivity remains critical to optimizing clinical outcomes for polymer materials.

### 3.2 Biodegradable metal implant

Biodegradable metals, including magnesium (Mg), iron (Fe), and zinc (Zn) based alloys, have been considered as potential candidates for orthopedic implants because they provide superior mechanical strength as internal fixation implants, degrade gradually, are biocompatible, and do not cause cytotoxicity to surrounding cells or tissues as defined by Zheng et al. [94].

Compared to bioresorbable polymers, biodegradable metals provide superior mechanical strength as internal fixation implants, especially in load-bearing sites. In general, cortical bone has a tensile strength of 105–114 MPa, a yield strength of 35–283 MPa, and an elastic modulus of 14–20 GPa [95]. Typically, osteoimplants with sufficient yield or tensile strength can effectively minimize fracture displacement and promote bone regeneration. In addition, a similar elastic modulus is also warranted for metal implants to avoid a “stress shielding effect' during the fracture healing period [51]. Among the biodegradable alloys, Fe-based alloys usually have some shortcomings, including a slow in vivo degradation rate, stable corrosion by-products, and poor biocompatibility with osseous tissues. In addition, Fe exists primarily in the blood environment and is not considered a potential orthopedic implant [62,96].

#### 3.2.1 Biodegradable magnesium alloys

**Fig. 4:**
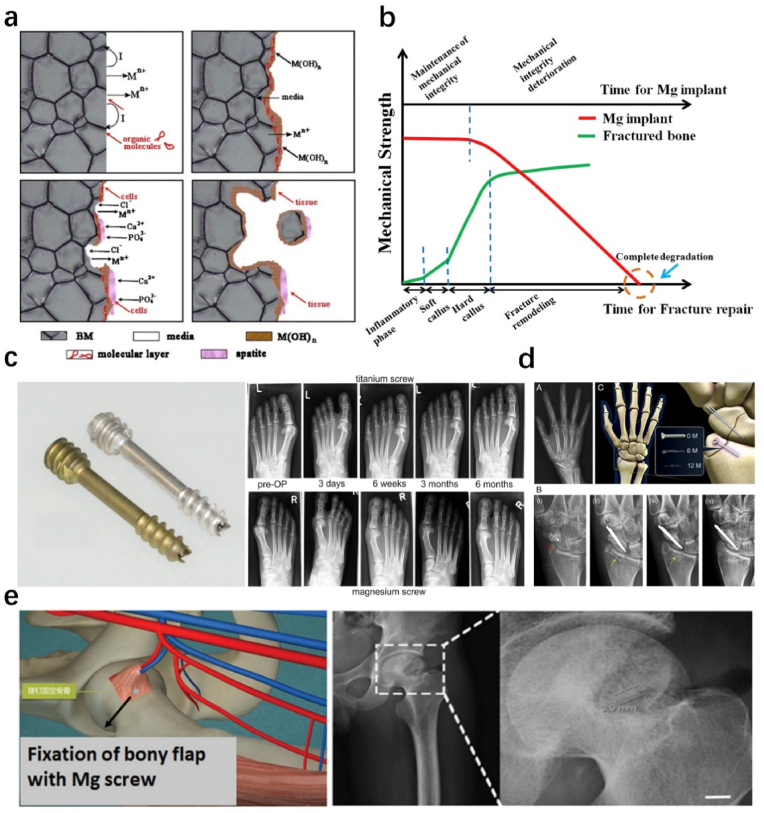
(a) Schematic diagrams illustrating the biodegradation process of Mg in the physiological environment. Reproduced with permission from Ref. [47]. Copyright 2014, Elsevier. (b) The relationship between mechanical strength and degradation behavior of an ideal orthopedic implant at different stages of bone regeneration. Reproduced with permission from Ref. [107]. Copyright 2016, Elsevier. (c) The commercial Mg-Y-RE-Zr alloy screws MAGNEZIX® provided by Syntelllix AG of Germany, and were used to treat a mild hallux valgus fracture in thirteen patients, with a 6-month follow-up for observation and assessment. Reproduced with permission from Ref. [105]. Copyright 2014, Springer. (d) The K-MET® screw developed by U&I Company based on Mg-5Ca-1Zn alloys, which were utilized to ix the distal radius fracture in 53 patients with a one-year follow-up for observation and assessment. Reproduced with permission [106]. Copyright 2016, National Academy of Sciences. (e) High-purity Mg screws used to fix the vascularized bony flap for hip preservation treatment. Reproduced with permission from Ref. [107]. Copyright 2016, Elsevier.

Mg is essential for the human body and is found in bones. The recommended daily intake is 375–500 mg [47,97]. Mg concentration in blood serum is 0.73–1.06 mM [97]. Mg has a wide distribution in human bones with an enrichment degree of around 53 % [98]. It also has a density of 1.74 g/cm^3^ and a elastic modulus of 45 GPa, similar to human bone. This avoids the stress shielding effect seen in traditional implants [47]. Mg is involved in over 300 enzymes essential for cell, nerve, and muscle function, and has no toxic effect on liver and kidney function [99]. Moreover, Mg is a very reactive metal with a standard electrode potential of −2.37 V (vs. SHE). As osteoimplants, Mg is a highly reactive metal with good compatibility with human tissues, which can reduce the inflammatory response and foreign body reaction caused by degradation by-products, as shown in Fig. 4a [47**]**. As illustrated by the Pourbaix diagram for Mg, the metal undergoes electrochemical dissolution into Mg^2+^ ions under typical physiological conditions (pH ∼7.4, E ≈ 0.78 V) [94]. This process triggers a gradual rise in local pH, theoretically enabling the sequential formation of MgCO_3_ and Mg(OH)_2_ layers. For Mg-based alloys, degradation initiates with the formation of Mg(OH)_2_, which reacts with chloride ions (Cl^−^) in bodily fluids to produce soluble MgCl_2_ and hydrogen gas [47]. Concurrently, Mg(OH)_2_ may interact with carbonate (CO_3_^2−^) or phosphate ions (PO_4_^3−^) to form MgCO_3_ or Mg_3_(PO_4_)_2_ (as shown in Table 1), respectively, depending on localized pH and electrolyte composition. The rapid H_2_ release risks subcutaneous gas cavity formation, potentially disrupting early-stage bone healing [100].

Mg-based devices were first used in 1878 for vascular ligation by Edward C. Huse [101]. In 1900, Payer used Mg wire as a suture, though it was brittle. He proposed using Mg in the musculoskeletal system, but preliminary animal results were not encouraging [102]. In 1906, Lambotte used a Mg plate and steel screws for internal fixation to treat post-traumatic tibia pseudarthrosis. However, the Mg plate was removed 8 days after surgery due to the formation of subcutaneous emphysema and limb swelling caused by rapid matrix degradation and hydrogen gas release [101]. This phenomenon was mainly attributed to the formation of galvanic corrosion between the Mg plate and the steel screws. Subsequently, Lambotte and Verbrugge used pure Mg plates and screws to treat supracondylar and intercondylar humerus fractures in children. They reported that more than 1 year after initial treatment, good fracture healing, restoration of joint function, complete degradation of the implants, and no apparent complications were observed [95,101]. On this basis, they continued to use pure Mg implants to treat fractures of the wrist, ankle, hand, clavicle, foot, and other bones [103].

Due to the rapid degradation of Mg, researchers turned their attention to Mg-based alloys. In 1948, Troitskii and Tisitrin added Cd to Mg and demonstrated the feasibility of Mg-Cd alloys as internal fixation plates or screws [104]. The alloy had osteoinductive properties and could stimulate callus formation. Serum Mg ion concentration was normal and no significant inflammatory response occurred. However, excessive gas generation was still a problem in clinical trials [101]. Based on these previous studies, the design and manufacturing of novel Mg osteoimplants have made great progress in recent years. In 2013, the German company Syntellix AG developed a novel Mg alloy screw (MAGNEZIX®) based on Mg-Y-RE-Zr alloys (as shown in Fig. 4c), which completed CE certification in the European Union. In addition, the screw was approved for the market in Singapore in 2016 [105]. The South Korean company U&I also developed the K-MET screw based on Mg-5Ca-1Zn alloys and obtained the authentication from the South Korean Food and Drug Administration [106], as shown in Fig. 4d. In China, Dongguan YiAn Technology Company has recently developed the “Biodegradable Mg Bone Fixation Screw', which has been approved by the Special Application Review of Innovative Medical Devices for Examination and Approval and is in clinical use. These pure Mg screws have demonstrated long-term efficacy (12 months) when used to fix autologous vascularized bone flaps in patients with femoral head avascular necrosis (Fig. 4e) [107].

To date, there are still some drawbacks that limit the widespread application of Mg-based implants: 1) The low mechanical properties (the highest tensile strength can reach up to ∼ 350 MPa) limit the structural design and application of implants in the load-bearing site [61]. For wide clinical application of WE43 magnesium alloy, the tensile strength of 250 MPa, far below the clinically commonly used titanium alloy (with a tensile strength of ∼895 MPa) or cobalt chromium alloy (with a tensile strength of ∼951–1220 MPa); 2) They have rapid degradation in vivo (0.36–1.58 mm/yr, tested by a rat femur model, 8–12 weeks) [108], easily losing mechanical support in the early stage, as shown in Fig. 4b. In addition, the large amount of hydrogen produced by the degradation of Mg alloy is also not conducive to bone healing [[109], [110], [111], [112]].

**Fig. 5:**
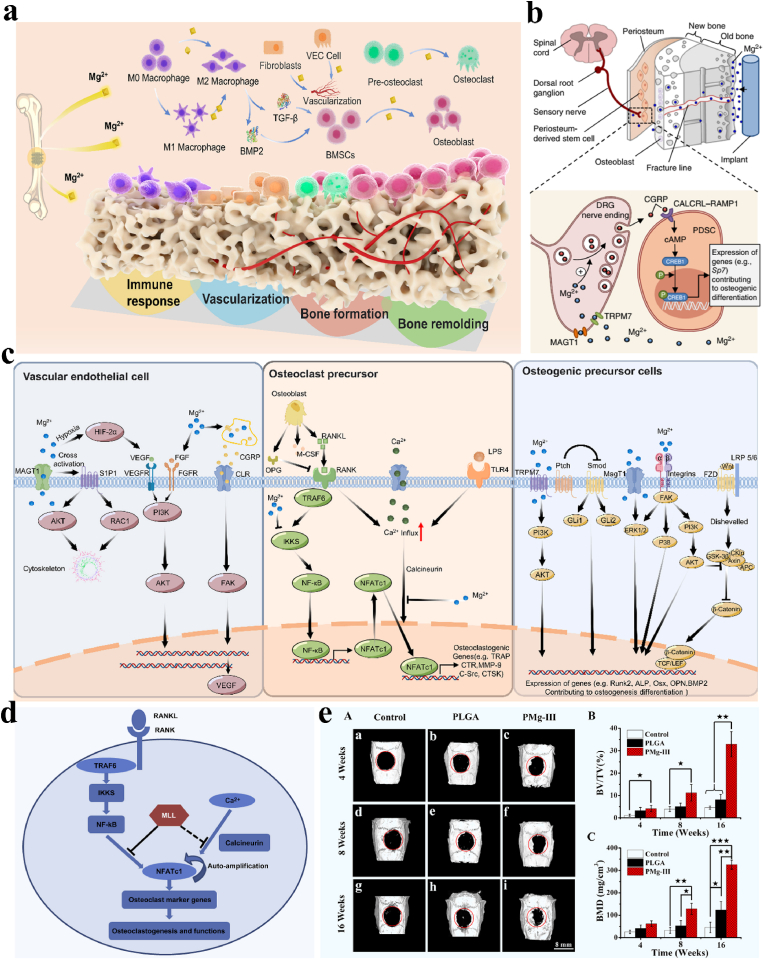
Cellular and molecular mechanisms revealing the potential effects of magnesium ions on bone repair. (a) A brief schematic diagram illustrationg the effect of Mg ions on surrounding cells involved in immune response, vascularization, bone formation and bone remodeling. Reproduced with permission from Ref. [113]. Copyright 2024, Elsevier.(b) A schematic illustration indicating that the activation of Mg-induced calcitonin gene-related peptide (CGRP) increases differentiation of periosteum stem cells (PSCs). Reproduced with permission from Ref. [114]. Copyright 2016, Springer Nature. (c) Potential biological functions of Mg^2+^ on cellular signaling pathways. Reproduced with permission from Ref. [113]. Copyright 2024, Elsevier. (d) The schematic diagram displaying magnesium leach liquor (MLL) reduces osteoclast differentiation and function by reducing NF-κB and NFATc1. Reproduced with permission from Ref. [117]. Copyright 2014, Elsevier. (e) Micro-CT pictures and accompanying analysis on control group and groups of PLGA and PMg size-III microspheres post operation for 16 weeks. ∗p < 0.05, ∗∗p < 0.01 and ∗∗∗p < 0.001. Reproduced with permission from Ref. [118]. Copyright 2018, Elsevier.

As potential internal fixation implants, the biological properties of the materials, especially for osteogenic activity, is the most important aspect to consider before clinical application. In recent years, Mg-containing biomaterials have demonstrated unique advantages in stimulating bone repair and remodeling. In general, the gradual release of Mg^2+^ due to in vivo degradation will stimulate the activity of peripheral cells, regulate the immune response, and improve the proliferation and differentiation of vascular endothelial cells and osteoblasts [113], as shown in Fig. 5a. Therefore, the biological performance of biodegradable Mg alloys is mainly correlated with the amount or distribution of released Mg^2+^ in vivo. The diffusion of released Mg^2+^ was found to occur via two different mechanisms: one through bone fracture gaps/lines or Harversian and Volkmann channels, and one from the bone marrow to the periosteum, which is densely packed with sensory nerve fibers and periosteal stem cells (PSCs). Qin et al. recently found that Mg can promote osteogenic differentiation and enhance fracture healing by stimulating the synthesis of neuronal calcitonin gene-related polypeptide-a (CGRP) in the ipsilateral dorsal root ganglia (DRG) and femoral peripheral cortex, as shown in Fig. 5b [114].

Additionally, Yoshizawa et al. looked into how Mg^2+^ affected the stimulation of human bone marrow stromal cells (hBMSCs) and found that a medium containing 10 mM Mg^2+^ could increase collagen type X protein and vascular endothelial growth factor (VEGF), which would in turn promote extracellular matrix (ECM) mineralization [115]. More importantly, they showed that hypoxia inducible factor-2a (HIF-2a) and peroxisome proliferator-activated receptor gamma coactivator (PGC)-1a jointly control Mg-induced VEGF. By triggering the PI3K/Akt signaling pathway, which includes the ion channel functional protein kinase, transient receptor potential cation channel subfamily M member 7 (TRPM7), a key catalyst, Mg^2+^ improves osteoblast adhesion, proliferation and differentiation, as shown in Fig. 5c [116]. From the perspective of osteoclastic mechanisms, Zhai et al. found that Mg leach liquor (MLL) prevented wear particle-induced osteolysis by preventing the activation of nuclear factor-κB (NF-κB), suggesting that Mg has an anti-osteoclastogenic effect (Fig. 5d) [117]. To date, the effect of Mg on the performance of in vivo osteogenesis has been thoroughly investigated. To investigate the effect of Mg^2+^ on the control of osteogenesis, Yuan et al. methodically prepared biodegradable microspheres (PMg) by embedding MgO and MgCO_3_ in poly(lactide-co-glycolide) (PLGA) microspheres at different weight ratios (1:0; 3:1; 1:1; 1:3; 0:1). PMg-III microspheres (MgO/MgCO_3_ in 1:1) showed greater new bone volume fraction (BV/TV) and bone mineral density (BMD) than PLGA and the control group according to the results of the in vivo critical size calvarial defect mode (Fig. 5e) [118].

In addition, Mg plays multiple roles in the nervous system, connecting bone, vasculature and the immune system to achieve the goal of functional bone regeneration and shows great potential as an osteo-implant. First, by facilitating the proliferation and differentiation of vascular endothelial and smooth muscle cells, the released Mg^2+^ can stimulate blood vessel development and improve blood flow to surrounding tissues, thereby promoting tissue regeneration [119]. By altering the PI3K/AKT and ERK1/2 pathways, Mg^2+^ may stimulate the production of key genes associated with vascular neoplasia, such as VEGF and basic fibroblast growth factor (bFGF) [120]. In terms of osteoimmunological responses, Mg has been found to reduce the M1 phase (supporting an inflammatory response) and promote macrophage polarization to the M2 phase (promoting osteoblast mineralization), suggesting that Mg also has an anti-inflammatory effect [121]. In the context of nerve regeneration, Mg^2+^ promotes axon regeneration and Schwann cell proliferation. Neurotrophic factors, such as nerve growth factor (NGF), are secreted by Schwann cells, which are essential components of the peripheral nervous system and help heal damaged nerves [122]. When Mg^2+^ is present in sufficient amounts, it promotes the growth of Schwann cells and the production of neurotrophic factors, which aids in axonal regeneration. As a result, Mg has a variety of effects on peripheral nerve regeneration, including neuroprotection, anti-inflammatory effects, and pro-regenerative effects (Fig. 5c). In addition, the rapid dissolution of Mg^2+^ from the sample surface has an antibacterial effect on *P. Aeruginosa* and *S. Aureus* [123]. Moreover, the local alkalinity near the surface and the generated Mg(OH)_2_ nanoflakes may also contribute to bacterial trapping, reactive oxygen species (ROS) generation, and bacterial apoptosis [[124], [125], [126]]. In fact, the biological effects of Mg on bone repair or bone remodeling are dose dependent. Typically, a reasonable amount of Mg^2+^ (5–15 mM) is conducive to bone regeneration and antibacterial activity, while high dose of Mg^2+^ may impair cell growth and surrounding tissues.

#### 3.2.2 Biodegradable zinc alloys

**Fig. 6:**
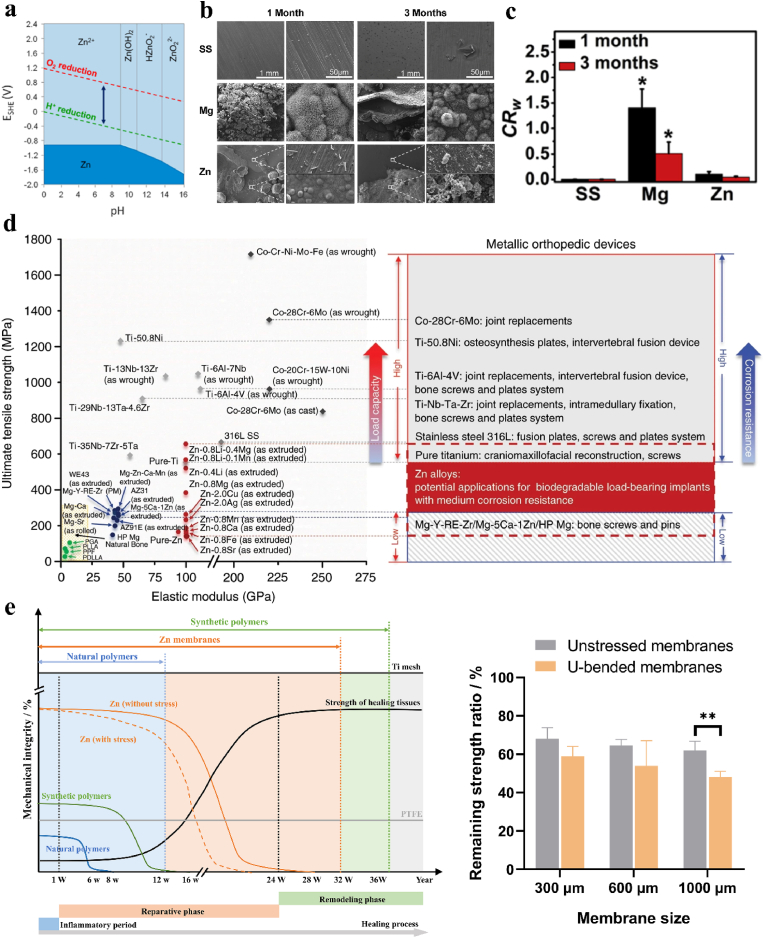
The corrosion behavior and mechanical properties of Zn-based alloys, (a) the Pourbaix diagram of Zn (the blue arrow indicates the biological standard reduction potentials at pH 7.4). Reproduced with permission from Ref. [137]. Copyright 2016, Elsevier. (b) the corrosion morphologies of stainless steel, Mg alloys and Zn alloys after immersion in Hanks' solution for 3 months, (c) the calculated corrosion rate after immersion tests. Reproduced with permission from Ref. [138]. Copyright 2019, Elsevier. (d) Summary of the mechanical properties of non-biodegradable and biodegradable materials for orthopedic scaffolds with potential clinical scenario. Reproduced with permission from Ref. [61]. Copyright 2019, Springer Nature. (e) A schematic diagram displaying the relationship between mechanical integrity and degradation time of Zn-based implants with or without bending stress, as well as the remaining tensile strength ratio of pure Zn membranes (different pore size). Reproduced with permission from Ref. [139]. Copyright 2024, Elsevier.

Compared to Mg-based alloy, zinc (Zn) and its alloys have recently been considered as potential candidates for orthopedic implants. Zn is one of the essential trace elements in human body, and the recommended daily intake of Zn ranges from 2 mg (infants) to 11 mg (adult males) [127]. In the human body, 85 % of Zn is found in the muscles and bones, and 11 % Zn is found in the skin and liver [128]. Zn is involved in nucleic acid metabolism, signal transduction, gene expression, apoptosis regulation, endocrine regulation and other physiological function of the human body [127]. Meanwhile, Zn is also involved in the synthesis, catalysis and regulation of more than 300 human enzymes [129]. The standard electrode potential of Zn is −0.76 V (*vs. SHE*), locating in the range of Mg (−2.37 V *vs. SHE*) and Fe (−0.44 V *vs. SHE*) [60]. Therefore, Zn has a more suitable in vivo degradation rate that matches the tissue repair process than Mg and Fe (Fig. 6b–c). Besides, in physiological environment, the cathode reaction of Zn is governed by oxygen absorption [130]. Thermodynamically projected surface oxides do not form an effective protective layer in the pH range of 7–10 because the lower cathodic reaction rates result in lower overall corrosion rates. Hence, as inspected in the Pourbaix diagram (Fig. 6a), Zn metal soaked in a physiological electrolyte pH of ∼7.4 will dissolve over time, as desired for orthopedic implant. Zn-based alloys degrade via Zn(OH)_2_ dehydration into ZnO, followed by carbonate-induced conversion to ZnCO_3_ (as shown in Table 1). While Zn^2+^ released at slower rates demonstrate dose-dependent osteogenic and antibacterial properties, excessive accumulation (>0.3 mM) suppresses osteoblast activity [131], necessitating precise degradation control.

In another aspect, the biomechanical performance of Zn-based alloy osteoimplants is a critical evaluation factor for juding the feasibility of materials as internal fixation implants. It has been suggested that load-bearing implants should have tensile strengths and elongations of at least 300 MPa and 15–18 %, respectively [132,133]. However, pure Zn (extruded state) usually present insufficient mechanical performance with tensile strength and elongation of around 166 MPa and 40 % [61]. Therefore, in recent years, great efforts have been devoted to developing novel Zn alloys by alloying method, additive manufacturing, powder metallurgy, etc. Yang et al. [61,132] successfully prepared different binary and ternary Zn alloys by adding human essential elements (Ca, Mg, Ag, Cu, Li and etc.), and the results indicated that the corporation of these elements would significant refine the microstructure, improve the mechanical properties and promote the osseointegration activity at bone/implant surface through multiple ions co-release, as desired implants as osteoimplants used at load-bearing sites. More importantly, Zn-based alloy implants present sufficient stress corrosion cracking(SCC) resistance (as shown in Fig. 6e), which may effectively avoid the subdent collapse of the osteoimplants during service and provide long-lasting mechanical support during service. Moreover, till now, it has been found that the mechanical strength of Zn-based osteimplants can be tailored by heat treatment, hot deformation, composite fabrication and etc. [[134], [135], [136]].

**Fig. 7:**
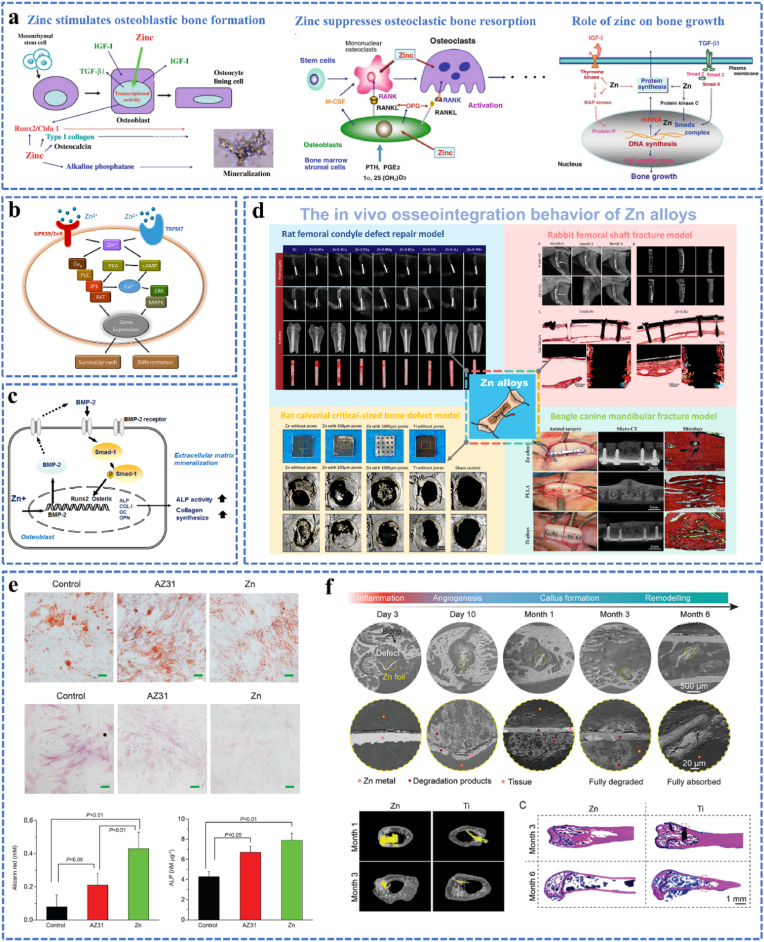
(a) The cellular response of Zn ions to surrounding bone environment, including that Zn promotes osteoblast cell development, proliferation, and mineralization; Zn prevents osteoclastic bone resorption by inhibiting the development of osteoclast-like cells induced by various bone-resorbing agents. The function of Zn in promoting bone formation is to increase protein synthesis in the translational phase by inducing aminoacyl-tRNA synthetase in osteoblastic cells. Reproduced with permission from Ref. [140]. Copyright 2009, Springer. (b) Zn^2+^ enters hBMSCs via two cell receptors, TRPM9 and GPR39, then activates cAMP-PKA signaling pathway and triggers intracellular Ca^2+^ response. Reproduced with permission from Ref. [141]. Copyright 2017, American Chemical Society. (c) Zn contributes to the bone morphogenetic protein-2 (BMP-2) signaling pathway in osteoblasts. Reproduced with permission from Ref. [141]. Copyright 2018, The Korean Nutrition Society. (d) the osteogenic activity of Zn-based alloys verified by rat femoral condyle defect repair model, rabbit femoral shaft fracture model, rat calvarial critical-sized bone defect model, and beagle canine mandibular fracture model. Reproduced with permission from Refs. [61,[147], [148], [149]]. Copyright 2019, Springer Nature. Copyright 2019, Elsevier. Copyright 2020, Elsevier. Copyright 2021, Elsevier. (e) ALP/ARS staining and activity in hBMSCs after 1 day of culture (scale bar = 20 μm). Reproduced with permission from Ref. [141]. Copyright 2017, American Chemical Society. (f) Biodegradation and osteogenesis of the Zn foils in mice femoral condyle during 6 months post-surgery. Reproduced with permission from Ref. [146]. Copyright 2023, Wiley-VCH GmbH.

As an osteoimplants, the celluar response of the materials to surrounding cells or tissues and corresponding molecular mechanisms are key factors influencing the clinical outcomes. Actually, Zn plays a significant role in bone microenvironment. A previous study conducted by Yamaguchi indicated that Zn could promote the differentiation of the osteoblasts and chondrocytes, while inhibiting osteoclast activity at the same time [140], as shown in Fig. 7a. Zhu et al. [141] recently studied the biocompatibility of pure Zn discs on human bone marrow mesenchymal stem cells (hBMSCs). It was found that pure Zn significantly promoted the mineralization of extracellular matrix and osteogenic differentiation of hBMSCs. Quantitative PCR showed significantly increased expression of bone-related genes (alkaline phosphatase, type I collagen and osteopontin). Zn^2+^ produced by degradation of pure Zn enters hBMSCs via two cell receptors, TRPM9 and GPR39, then activates cAMP-PKA signaling pathway and triggers intracellular Ca^2+^ response (Fig. 7b), and finally promotes osteogenic differentiation and mineralization by activating MAPK to up-regulate the expression of related genes (Fig. 7e). More importantly, Zn stimulates gene expression and activates tRNA synthase to increase protein synthesis in osteoblasts. By boosting the cells' DNA content, it simultaneously encourages osteoblast osteogenesis and mineralization [142,143]. Besides, insufficient cellular Zn influences BMP-2 signaling, which in turn impacted the production of bone marker genes and proteins, as well as bone-specific transcription factors (Runx2 and Osteix), ultimately influencing osteoblast differentiation (as shown in Fig. 7c) [144]. Therefore, till now, the osteogenic activity of Zn and Zn-based alloys have verified by in vivo rat femoral condyle defect model, rabbit femoral shafte fracture model, rat calvarial critical-sized bone defect model, beagle canine mandibular fracture model, and etc. (as illustrated in Fig. 7d). Actually, the biocompatibility of the material is closely related to the concentration of Zn ions produced by material degradation, low concentration of Zn ions can promote bone formation and mineralization, while high concentration produces the opposite effect. Murni et al. [145] studied the effect of pure Zn and Zn-3Mg alloys on normal human osteoblast cells (NHOst). The study found that the cultured osteoblasts showed good cytocompatibility at concentrations below 0.5 mg/mL, but at concentrations above 1.0 mg/mL, the material extracts showed grade 3 cytotoxicity. Besides, osteoblasts cultured with 0.75 mg/mL of pure Zn and Zn-3Mg alloy extracts showed grade 2 cytotoxicity on Day 1. With the prolongation of culture time, the survival rate of the Zn-3Mg alloy group was improved, while the pure Zn group still showed significant cytotoxicity. Recently, Yang et al. [146] tracked the biodegradation process of pure Zn foils through a mouse femoral condyle defect repair model, the results indicated that Zn implant with a suitable Zn ion release appears to have a more prominent osteogenic impact than the Ti implant, as seen in the staining results (red dashed circles at Month 3, as shown in Fig. 7f). Consequently, all of the aforementioned results point to Zn's significant function in bone metabolism and its enormous potential as a osteoimplant.

**Fig. 8:**
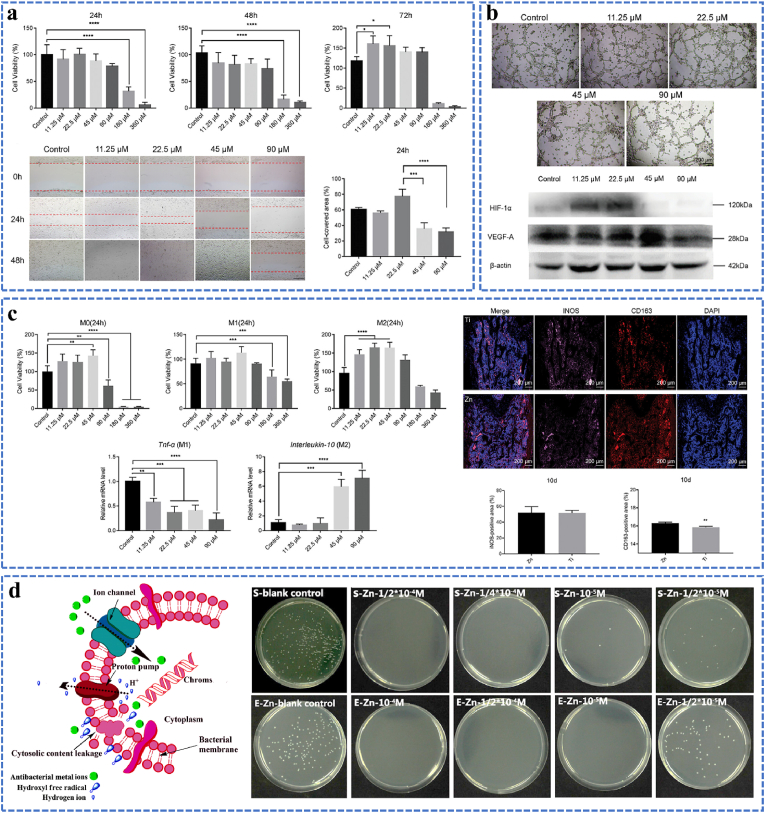
(a) Impact of varying amounts of Zn extraction on HUVECs migration and viability in vitro, (b) Effect of Zn extracts on Tube formation of HUVECs and Western Blot of angiogenesis-related genes (Hif-1*α* and Vegf-a), (c) Effect of Zn and degradation on macrophages; effects of varying Zn extraction concentrations on macrophage polarization and cell survival in vitro; the expression of typical markers associated with macrophage polarizations (Tnf-*α*, M1; Interleukin, M2) and the cell survival of macrophages (M0, M1, M2) in response to Zn extracts at varying doses; On Day 10 following surgery, macrophages were stained with immunofluorescence (iNOS, CD163, DAPI). Reproduced with permission from Ref. [150]. Copyright 2023, Wiley-VCH GmbH. (d) The antibacterial behavior of Zn^2+^ against S. aureus and E. coli, and corresponding antimicrobial mechanism. Reproduced with permission from Ref. [151]. Copyright 2015, American Chemical Society.

After an injury, bone healing is a carefully regulated process that begins with hematoma formation, blood clotting, and a pro-inflammatory phase. Therefore, the vascularization and immune regulation process of the implant should be considered. On the basis of in vitro results, at concentrations of the Zn extract above 90 μM, human umbilical vein endothelial cells (HUVECs) proliferation is greatly suppressed and hazardous, whereas at values below 90 μM, it is significantly encouraged (as shown in Fig. 8a) [150]. Besides, it can also be observed that the migration inhibition of HUVECs was evident when the Zn extraction concentration exceeded 22.5 μM. The inhibition of migration became more noticeable as the concentration rose. On the other hand, no discernible inhibition was noted at Zn extraction concentrations lower than 22.5 μM. The extension of tubule length and the increased creation of cross-linking sites on the stromal gel indicate that 22.5 μM is the most notable concentration for improving tube formation in endothelial cells. More importantly, higher expression of Hif-1*α* and Vegf-a was linked to an increase in the Zn leaching solution concentration (up to 22.5 μM), as illustrated in Fig. 8b. Regarding the effect of Zn extracts on macrophage behavior, it can be inspected from Fig. 8c that M1 macrophage proliferation was not significantly impacted by Zn extracts with concentrations ranging from 11.25 to 45 μM, however M0 and M2 macrophage proliferation was enhanced. Additionally, incubating macrophages from M0 toward the M2 phenotype was triggered by extracts containing 11.25–90 μM Zn. The Zn extract encouraged the macrophages to primarily specialize toward the M2 phenotype, according to a subsequent qPCR examination of macrophage phenotypes [150]. Furthermore, after 10 days post-operation, the in vivo staining results indicated that the Zn-implant group had a considerably larger expression of CD163 than the Ti-implant group, while both groups displayed a similar expression of inducible nitric oxide synthase (iNOS). These results illuminate its possible uses and offer crucial insights into the biochemical effects of Zn on bone repair.

In clinical studies, orthopedic implant infections are a prominent cause of implant failure in vivo. Hence, the implants with sufficient antibacterial activity is warranted in orthopedic surgeries. Fortunately, Zn^2+^ has a wide range of antibacterial properties, and the amount of Zn^2+^ released in vivo largely determines the bacteriostatic activity of Zn alloys. The antibacterial activity of Zn^2+^ can generally be categorized as follows [[151], [152], [153]]: (1) Bacteria have a negative charge on their surface, while Zn^2+^ has a positive charge. Because of coulombic force, Zn^2+^ will adhere to the surface of bacteria, damaging the cell wall and releasing molecules like lactate dehydrogenase (LDH) [151], as shown in Fig. 8d. (2) Zn^2+^ interacts with proteins or anionic groups in bacteria, denaturing proteins and reducing cell synthase activity, which prevents bacteria from growing and reproducing normally. (3) When Zn^2+^ reacts with DNA, it damages parts of the cell's functioning systems, prevents the bacteria from metabolizing normally, and eventually kills the bacterium. Additionally, Zn alloy-induced micro-galvanic corrosion acts as a proton pump, continuously transporting internal H^+^ to the extracellular environment. As H^+^ transfer rises, adenosine triphosphatase (ATP) synthesis is inhibited, which leads to additional bacterial death. Therefore, the amount of liberated Zn^2+^ largely determines the antibacterial efficacy of Zn alloy. Ning [146,151] examined the cell proliferation of fibroblast cells L929 and the antibacterial rate against *S. aureus* and *E. coli* in the presence of different Zn^2+^ concentrations. The findings indicate that at concentrations below 10^−4^ M of Zn^2+^, the cell growth rate was higher than 80 %, and the minimal bactericidal concentration (MBCs) of Zn^2+^ is roughly 10 μM. In order to eradicate harmful microorganisms and encourage bone repair at the same time, it is therefore very desirable to balance the effects of Zn on bacteria and mammalian cells.

#### 3.2.3 Biodegradable iron alloys

Compared to Mg- and Zn- based alloy, degradable iron (Fe)-based alloys show conditional promise for pediatric orthopedic applications, balancing advantageous mechanical compatibility with juvenile bone (ductility >40 %, UTS 200–250 MPa) and evolving corrosion-enhancement strategies [154]. While advanced Fe-Mn/Pd alloys achieve accelerated degradation rates (300–400 % faster than pure Fe) through galvanic mechanisms and demonstrate clinically acceptable biocompatibility (>90 % in juvenile models) and osteointegration (68 ± 9 % BIC ratio) [62,96,155,156], critical limitations remain. These include the risk of inflammation from micron-scale corrosion debris, MRI interference from residual ferromagnetism, and inadequate load capacity in porous designs [62]. Current studies focus primarily on polymer-coated Fe-Mn scaffolds that meet degradation resistance benchmarks for non-load-bearing pediatric applications such as cranial fixation [156], although full structural deployment awaits advances in nanoscale laminate fabrication and ion sequestration technologies to resolve the plasticity/degradation paradox.

When it comes to the biological activity of metallic materials, the ion release rates of biodegradable metals must be emphasized, which must be aligned with age-specific daily intakes to avoid toxicity. For example, Mg-alloy degradation releases Mg^2+^ ions, which, while essential for bone metabolism, may exceed pediatric thresholds (30–75 mg/day for children vs. 400 mg/day for adults) if corrosion rates are unmodulated [157]. Similarly, Zn-alloy rapid degradation in physiological environments risks surpassing tolerable limits (3–8 mg/day for children vs. 11 mg/day for adults), potentially causing gastrointestinal or immunotoxicity. Fe alloy slower corrosion may reduce acute risks, but cumulative iron overload remains a concern in repeated applications. Recent studies emphasize that surface coatings and alloy design (e.g., Mg-Zn-Ca alloys) can modulate ion release kinetics to align with pediatric metabolic capacities [157]. However, standardized in vivo models for age-dependent degradation profiles are still lacking, underscoring the need for pediatric-specific regulatory frameworks.

## 4 Application of absorbable implants in pediatrics

**Fig. 9:**
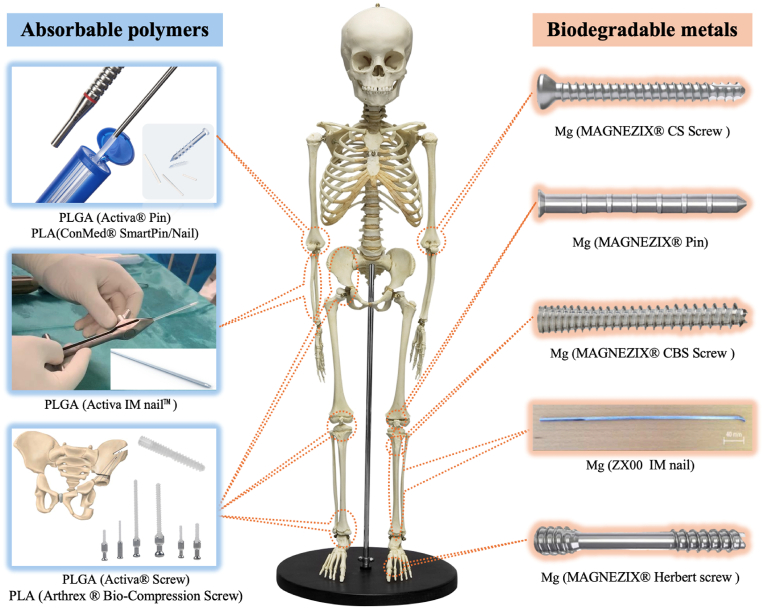
Several representative applications of absorbable implants for pediatrics in different parts of body.

In recent years, the use of internal resorbable implants has gradually increased in the pediatric population, with reports of fixation of elbow, forearm, hip, knee, ankle, and foot fractures published in the literature [[158], [159], [160], [161], [162]]. The main forms of internal implants include pin, screw and intramedullary nail. Several representative applications of polymer and metal alloy resorbable implants in pediatrics at various sites of the skeleton are shown and summarized in Fig. 9.

### 4.1 Pins

**Fig. 10:**
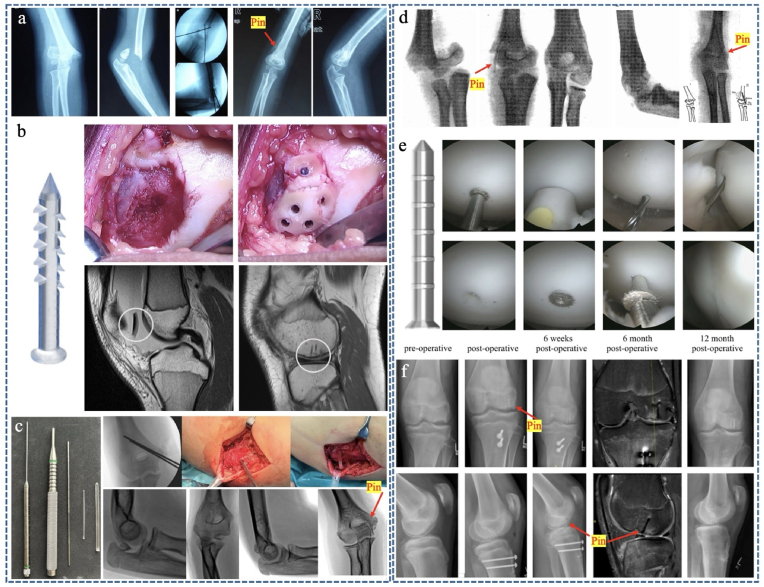
(a) X-ray showed Gartland type III supracondylar fracture of the humerus in a 5-year-old girl, once reduction of the fracture and location of the pins were confirmed satisfactory, the K-wires were replaced with the GRANDFIX™ PDLLA bioabsorbable pins through the primary pin tracks. Reproduced with permission from Ref. [164]. Copyright 2011, Springer. (b) Intraoperative images of two pieces of the osteochondral fracture (OCF) fixated with six bioabsorbable PLA SmartNails, Preoperative MRI of the OCF fragment and postoperative MRI showing OCF healed. Reproduced with permission from Ref. [171]. Copyright 2022, SAGE. (c) Medial epicondyle avulsion fractures in an 11-year-old girl were reduced and stabilizated with K-wires: under fluoroscopy; If the wires were in an optimal position, then they were replaced with PLGA biodegradable pins. Reproduced with permission from Ref. [168]. Copyright 2022, Wolters Kluwer Health. (d) Lambotte and Verbrugge shows a supracondylar humerus fracture of a child fixated using a magnesium pin, and the early onset of subcutaneous gas formation can be seen. Reproduced with permission from Ref. [104]. Copyright 2010, Elsevier. (e) Arthroscopic insertion of the pin and (f) Fixation of a displaced osteochondral fragment of the lateral femur condyle after patella dislocation with 3 pins and realignment of the patella using the Elmslie-Trillat procedure in a 16-year-old boy. Reproduced with permission from Ref. [174]. Copyright 2021, SAGE.

Bioabsorbable polymer pins have been used in pediatric orthopedic trauma for over 30 years, and several early studies have shown that PGA, PLA, or PLGA pins are comparable to metallic titanium or stainless steel pins with fewer complications (Fig. 10a–c) [[163], [164], [165]]. The use of the degradable metal pin in pediatric orthopedics dates back to the 1930s, when Lambotte and Verbrugge first attempted to treat supracondylar fractures by placing a low-volume Mg pin extra-articularly in four children. All healed without complications, with good joint function and no pain (Fig. 10d) [104].

In a prospective randomized clinical trial, Hope et al. [163] compared biodegradable PGA pins and Kirschner wires for fixation of displaced elbow fractures in children (n = 24). The Kirschner wire group had complications, including infection (n = 3) and heterotopic ossification (n = 1), and required hardware removal under general anesthesia. The PGA pin group had only one complication, avascular necrosis and premature fusion of the medial epicondyle.

Absorbable PLA pins have also been widely used in the treatment of displaced fractures in children, achieving the same healing results as traditional metal internal fixation and avoiding a second surgery for removal, but also fewer pin track infections, including supracondylar humerus (Fig. 10a) [164], radial neck [166] and internal epicondyle of humerus (Fig. 10c) [167].

Absorbable PLGA has also been compared with K-Wires and no differences in complication rates have been found in children with medial humeral epicondyle fractures [168], lateral condyle fractures [158] and distal forearm or metaphyseal radial fractures [169]; they have the advantage that hardware removal is not needed.

Bioabsorbable polymer pins have also been widely used in pediatric arthroscopic surgery with satisfactory clinical results, including including osteochondritis dissecans (OCD) [170] of the knee and osteochondral fracture (OCF) (Fig. 10b) [171]. Jungesblut et al. [172] reported that 19 adolescents with displaced OCD or OCF were fixed with MAGNEZIX® pins, which provided high stability and rapid healing at an 11-month follow-up. (Fig. 10e–f).

Detailed materials used, treatment methods and clinical results are shown in Table 2 and Fig. 10.

**Table 2:** Relevant studies with different types of resorbable pins for pediatric use.

Year/Journal	Study model	Implant type and material	Methodology and research questions	Key findings
1991, JBJS(B) [163]	Prospective pilot study	• PGA (polyglycolic acid) • Stainless steel (K-wires)	• Displaced elbow fractures in children. • Evaluated fracture union, range of movement, physeal appearance and complications. • Follow-up after at least 12 months.	• Fractures union with full function in all cases. • K-wires group with three infection, one ossification and nine required inplants removal under general anesthesia. • PGA Pins group without complications but one medial epicondyle necrosis and premature fusion.
2011, Int Orthop [164]	Clinical pilot study	• PLA (GRANDFIX™, Japan)	• Gartland-III supracondylar fracture of humerus. • After reduction of the fracture, K-wires were replaced with PLA pins, evaluated by MEPS and Flynn Criteria. • Followed up 24–36 months.	• MEPS: 94.6 % excellent and good outcome • Flynn Criteria: all excellent results but one not. • PLA pins avoid a second operation to remove.
2010, JPO [165]	Retrospective pilot study	• PLGA (SmartNail, FL) poly-96L/4D-lactide copolymer	• Juvenile osteochondritis dissecans (OCD) lesions • Arthroscopic retrograde drilling and put-in inplants • Follow-up 39.6 months	• All patients have good-to-excellent outcomes. • No significant change in 1 patient. • Loose bodies with no interval healing in 1 patient.
2015, JPO [161]	Retrospective pilot study	• PLA (NEOFIXPin, Japan)	• Unstable juvenile osteochondritis dissecans lesions. • Fixed with PLA pins under arthroscopy. • Mean follow-up for 3.3 years.	• Radiographic 32 of 33 lesions healed. • Hughston's criteria: 4 good and 1 poor. • PLA pins improved clinical outcomes and healing rates.
2016, JPO [166]	Retrospective pilot study	• PLA (SmartPin®, ConMed)	• Radial neck fractures in children. • Open reduction and fixed with SmartPins. • Postoperative follow-up averaged 9.2 months	• No evidence of physeal closure or avascular necrosis • No cases complicated by local inflammatory reactions. • Radioulnar synostosis after open surgery is still a concern.
2020, Medicine [167]	Retrospective controlled study	• PLA (Pins) • Metallic (K-wires)	• Medial humeral epicondylar fractures in children. • Fractures reduced and fixed with K-wires or PLA pins. • Followed up for over 12 months.	• K-wires group developed 4 pin-track infections. • PLA pins group with higher Broberg and Morrey score and better functional outcome than K-wires group.
2021, Cartilage [170]	Retrospective multicenter study	• PLA (SmartPin®, Edison, NY)	• Osteochondritis dissecans (OCD) in juvenile patients • Drilling and arthroscopic fixation with PLA pins. • An average follow-up of 6.6 years.	• Healing of the lesion in 36 out of 40 patients (90 %). • No infection, knee stiffness or other complication. • PLA pins provides good to excellent outcomes for OCD.
2022, JCO [171]	Retrospective case series	• PLA (SmartPins/Nails, ConMed Linvatec)	• Patellar dislocation with osteochondral fractures (OCF) • Medial patellofemoral ligament reconstructions and simultaneous OCF fixation with PLA pins. • Average follow-up was 2.6 years.	• OCF fixation with PLA pins success in 98 %. • OCF in adolescents with patellofemoral instability can be effectively treated with resorbable pins fixation and medial patellofemoral ligament reconstruction.
2022, Medicine [168]	Retrospective cohort study	• PLGA (Activa Pin®) + PDS (polydioxanone sutures)	• Medial humeral epicondyle fractures. • Stabilized with PLGA Pin and tension band PDS. • Average follow-up 34 months.	• All fractures healed with complete ROM of the elbow. • PLGA pins with absorbable sutures are a good alternative treatment of medial epicondyle humeral fracture.
2022, BMC MD [169]	Retrospective multicenter study	• PLGA (Activa®Pin) • Stainless steel (K-wires)	• Distal forearm or metaphyseal radial fractures. • K-wires either buried under skin or left outside the skin. • Follow-up after at least 1.5 years.	• Complication rate of PLGA pins group significantly lower than K-wires buried group or K-wires left outside group. • No second surgery in PLGA pins group so cost saving.
2024, Injury [158]	Retrospective controlled study	• PLGA (ActivaPin™) + PDS ® • Metallic (K-wires + tansion band wire)	• Lateral condyle fractures in paediatrics. • Open reduction and fixed with PLGA pins or K-wires combined with tension band systems. • Follow-up more than one year.	• No difference between groups in number of complications. • PLGA pins not need second intervention with significant cost-effectiveness.
2010, Acta Biomater [104]	Literature review report	• Mg nail (pin)	• Lambotte and Verbrugge first attempted to treat supracondylar fractures of humerus in four children. • Reduced the fracture and inserted with a Mg pin.	• All fractures healed without complications except the gas cavities, with good joint function and no pain. • Gas cavities disappeared after several weeks.
2021, Cartilage [172]	Prospective cohort study	• Mg (MAGNEZIX® Pin)	• Unstable OCD or displaced OCF in adolescents. • Arthroscopic confirmation and fixed with Mg Pins. • Follow-up of 11 ± 4 months.	• Mg pins provide high stability after fixation of unstable OCDs and displaced osteochondral fragments leading to uncomplicated and timely healing.
2021, Injury [173]	Retrospective case series	• Mg (MAGNEZIX® CS or CBS screws) • Mg (MAGNEZIX® Pin)	• 89 patients: 38 treated by osteosynthesis, 18 osteotomy and 33 osteochondral refixation. • The mean follow-up duration was 8.2 months.	• One broken pin (1/91) led to implant migration and revision surgery. • Mg Pins showed a low rate of revision surgery with good results for fracture fixation, osteotomy and osteochondral defect refixation.

### 4.2 Screws

**Fig. 11:**
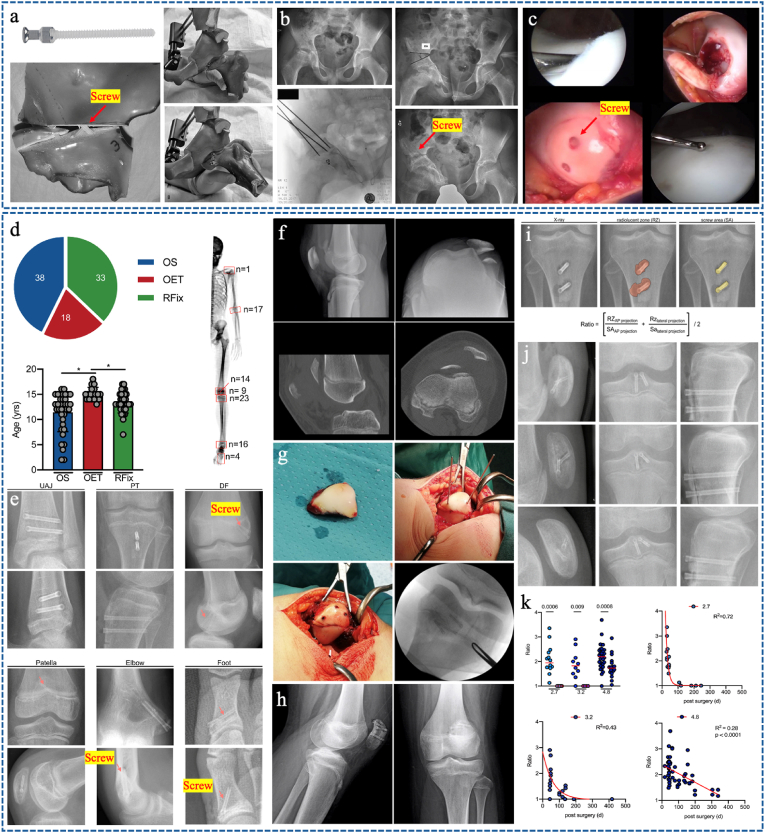
(a) Test stability of triple innominate osteotomy internally fixed with 2–3 bioabsorbable screws or steel screws. Reproduced with permission from Ref. [175]. Copyright 2007, Wilkins. (b) A 10.5-year-old boy with Legg-Calvé-Perthes disease conduct a triple osteotomy fixed with resorbable PLGA screws. Reproduced with permission from Ref. [160]. Copyright 2021, Wolters Kluwer Health. (c) A17-year-old boy diagnosed with osteochondritis dissecans of the lateral aspect of the trochlea and underwent open reduction and fixation with 2 bioabsorbable screws, second-look arthroscopic demonstrated fully healed. Reproduced with permission from Ref. [179]. Copyright 2019, SAGE. (d–e) Overview of the presented surgeries and the included patient cohort and implants at the different anatomical localizations. Reproduced with permission from Ref. [173]. Copyright 2021, Elsevier. (f–h) X-ray and CT showing a free osteochondral fragment along with lateral dislocation of the patella. Reproduced with permission from Ref. [180]. Copyright 2021, SAGE. (i–k) Evaluation of the radiolucent zone (RZ)/screw area (SA) ratio by use of standard radiographs to assess gas release from MAGNEZIX® CS 2.7 mm, CS 3.2 mm and CSC 4.8 mm Reproduced with permission from Ref. [182]. Copyright 2022, Springer.

Screws have better tensile and compressive strength than pins, making them suitable for more complex mechanical applications. Adamczyk et al. [175] found that bioabsorbable screws had similar stability to steel screws in patients undergoing triple pelvic osteotomy, but also had the advantage of not requiring a second surgery for screw removal (Fig. 11a).

Subsequently, Hedelin et al. [160,176] widely used degradable polymer screws for pelvic Salter's innominate osteotomy and triple pelvic osteotomy in patients with Developmental Dysplaia of the Hip (DDH), Legg-Calvé-Perthes Disease (LCPD), or Down syndrome (Fig. 11b), and reported that all osteotomies healed without complications. Hedelin et al. [177] used MRI to evaluate the resorption of PLGA screws used in pediatric pelvic osteotomies. They appeared to be resorbed and replaced by solid bone in most cases, although this took at least 2 years and minor reactions were seen in the adjacent bone.

Degradable polymer screws have been used in pediatric fractures, including open reduction and fixation of medial epicondylar fractures in adolescents, with results comparable to conventional screws, but with fewer complications and no need for implant removal [168,178]. Schlechter et al. [179] also used PLA screws to treat adolescent OCD and OCF with satisfactory results (Fig. 11c).

Magnesium screws were used for pediatric osteosynthesis, osteotomy, and osteochondral fixation. The most widely used magnesium alloy screws were manufactured by MAGNEZIX, Syntellix AG, Hannover, Germany [173,[180], [181], [182]]. Stürznickel et al. [173] reported on 64 children who received magnesium alloy screws for shoulder, elbow, knee, ankle, and foot pathology. They achieved adequate bone healing and showed a low rate of revision surgery (Fig. 11d–e). Baldini et al. [181] used magnesium screws versus K-wires in displaced medial epicondyle fractures and found comparable results with lower rates of nonunion, infection, and adverse reactions; the same authors reported similar results when using magnesium screws to fix epiphyseal fractures and OCD lesions (Fig. 11f–h) [180]. Recently, Delsmann et al. [182] retrospectively analyzed the radiolucent zones after implantation of magnesium-based compression screws of different diameters (2.7 mm, 3.2 mm, and 4.8 mm) in 29 children and adolescents undergoing fracture fixation, osteotomy, or OCD fixation. They found a significant linear and slower decrease in radiolucent zones with the ceramic-coated 4.8 mm screws, but a faster decrease during the first weeks with the uncoated 2.7 mm and 3.2 mm screws (Fig. 11i–k). At present, there are still few reports on the degradation rate of materials in children and the difference between children and adults, only found Marek R et al. [183] demonstrate that degradation rate of ZX00-implants in sheep ranges between 0.23 and 0.75 mm/year, and the highest degradation rates were found in the epiphysis. We sequentially concluded that the degradation rate of degradable alloys in children, especially around epiphyseal, is likely to be faster than that in adults.

Detailed materials used, treatment methods and clinical results are shown in Table 3 and Fig. 11.

**Table 3:** Relevant studies with different types of screws made of resorbable materials use in pediatrics.

Year/Journal	Study model	Implant type and material	Methodology and research questions	Key findings
2007, JPO [175]	Biomechanical study	• PLA (Inion Inc, Finland) • Stainless steel (Synthes Inc)	• Compare the biomechanical stability of triple innominate osteotomies fixed with either three 4.5-mm PLA or stainless steel screws.	• No differences for fragment displacement or construct stiffness. • Bioabsorbable screws have similar stability for triple osteotomies and also advantage of not need for removal.
2019, OJSM [179]	Retrospective case series	• PLA (Bio-Compression Screws Arthrex)	• Osteochondral lesions (OCL) in the adolescent knee • Arthroscopic fixation of PLA screws • Follow-up at least 2 years.	• PLA screws appears to be a safe and efficacious treatment for adolescent knee OCL with good functional outcomes and a low revision rate at long-term follow-up.
2019, JCO [176]	Retrospective case series	• PLGA (Activa Screw, Finland)	• DDH or LCPD in children • Salter pelvic osteotomy and fixed with PLGA screws. • Follow-up at least 6 months.	• PLGA screws provided sufficient stability and caused no local reactions • Resorbable implants gave the surgeon a wider range of possible screw placements and avoided the need for implant removal.
2020, JOSR [177]	Retrospective case series	• PLGA (Activa Screw, Finland)	• Analysis the MRI after Salter or Triple pelvic osteotomy and fixed with PLGA screws. • Eight parameters relating to screw resorption, local reactions and re-formation of bone were interpreted.	• The screw canals were >90 % replaced with solid bone after 2–4.5 years. • No soft tissue reactions but small bone cysts observed in 3/18 MRIs. • PLGA screws in the pediatric pelvis appear to be resorbed and replaced with solid bone in most cases but this process takes at least 2 years.
2021, JPO [160]	Retrospective case series	• PLGA (Activa Screw, Finland)	• DDH, LCPD or Down syndrome • Triple pelvic osteotomy (TPO) and fixed with PLGA screws. • Follow-up at least 20 weeks	• PLGA screws provides sufficient stability and appears to be a promising alternative to traditional TPO. • Resorbable screws enable the surgeon to place implants with more degrees of freedom and no need implant removal.
2022, Medicine [178]	Retrospective controlled study	• TMC Bioabsorbable screw (BS) • Cannulated lag screw (CLS)	• Medial epicondylar fractures in adolescents • Open reduction and fixed with BS or CLS. • Follow-up for 6 months	• No significant difference of elbow ROM or MEPS between 2 groups. • BS group (0 %) has lower implant prominence than CLS (33.3 %). • BS group has the advantage of not needing implant removal.
2021, JCO [180]	Retrospective case series	• Herbert-type Mg screws (MAGNEZIX; Hannover, Germany)	• Epiphyseal fractures or osteochondritis dissecans (OCD). • Reduction and fixed with Herbert-type Mg screws • Follow-up for three or more months.	• Healing was achieved in all the procedures. • No implant-related adverse reaction or need second surgical procedure. • Mg screws used in children is low but a promising stable fixation with good clinical and radiological results and no adverse events.
2021, Injury [173]	Retrospective case series	• Magnesium (MAGNEZIX® CS or CBS screws) • Magnesium (MAGNEZIX® Pin)	• 89 patients: 38 treated by osteosynthesis, 18 osteotomy and 33 osteochondral refixation. • 64 patients treated with Mg screws • Mean follow-up of 8.2 months.	• Mg CS or CBS screws were promising for fracture fixation, osteotomy and osteochondral defect refixation in children and adolescents, with stable fixation achieved in all cases. • No general differences in outcomes among the skeletal sites.
2023, JCO [181]	Retrospective controlled study	• Herbert-type Mg screws (MAGNEZIX®, Syntellix AG) • K-wires	• Medial epicondylar fracture of humerus • Open reduction and fixed with Mg screws or K-wires. • Follow-up at least 2 years.	• Mg screws showed comparable results as K-wires, potentially with a lower incidence of non-union and infection. • No adverse reactions and no need for a second surgical intervention of implant removal in Mg screws group
2023,AOTS [182]	Retrospective controlled study	• Compression Mg screws (MAGNEZIX® CS 2.7 mm, CS 3.2 mm, CSC 4.8 mm; Syntellix AG)	• Osteotomy, fracture or osteochondral refixation. • The ratio of radiolucent zone of the screw was evaluated to assess implant degradation at two follow-up visits (i.e., after 6–8 weeks and 12–24 weeks).	• The ceramic-coated 4.8 mm screws presented a significant linear and slower decrease of radiolucent zones. • Radiolucent zones are a common phenomenon of implant degradation and without implant failure or affected implant function.

### 4.3 Intramedullary nails

**Fig. 12:**
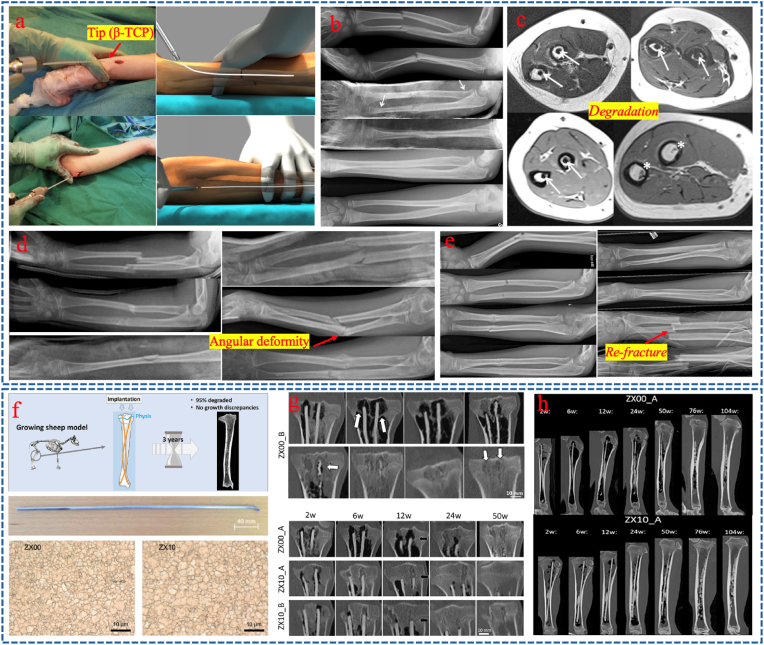
(a) Biodegradable intramedullary nail of PLGA with a fluoroscopy positive tricalcium-phosphate (β-TCP) marker (white arrow) introducing the implant into radius and ulna. (b) A 9-year-old boy with forearm bones stabilized by means of Activa IM nail. (c) MRI demonstrated the biodegrading process of PLGA IM nail in 3 months, 12 months and two-year later. (d) Two patients (14 years old boy and 13 years old girl) treated with the study implants (BIN) suffered from sudden implant failure, the bones were found to be angulated and then stabilized with plate fixation. (e) Another two patients (10 years old girl and 8 years old boy) suffered from an unstable diaphyseal forearm fracture after both bone union. Reproduced with permission from Ref. [185]. Copyright 2018, Elsevier. (f) ESIN made of XHP ZX10 with a shovel-formed tip and Microstructure of ZX00 and ZX10. (g) CT images of ZX10 and ZX00 in animals showed gas produced, but did not affect physis growth and tibia lengthening; (h) One ZX10 in animal and One ZX00 in another are shown until 104 weeks after implantation. Reproduced with permission from Ref. [189]. Copyright 2023, Elsevier.

Only one polymer resorbable intramedullary nail (RESIN), the Activa IM Nail™ made of PLGA, has been used to date for the treatment of non-weight bearing ulnar and radial fractures in children [159,[184], [185], [186], [187], [188]]. Compared to Titanium Elastic Nails (TEN), the RESIN requires preparation of the medullary canal after fracture reduction with an implant-specific dilator tool and then replacement with the degradable polymer nails, which is a more complicated procedure that prolongs the surgical procedure [188]. Because PLGA implants are radiolucent, with only the tricalcium phosphate (beta-TCP) tip visible on radiographs, it is not easy to assess the position of the intramedullary nail, and MRI is required to assess implant degradation (Fig. 12a–c). In addition, Korhonen et al. [185] reported that angular deformation occurred after fixation due to the lower strength and elastic modulus of the polymer material compared to TENs (Fig. 12d). In addition, the same authors reported that 2 of 38 cases had secondary fractures at or near the original fracture site, and it is possible that the acidic polymer environment could negatively affect fracture healing (Fig. 12e) [185]. Multi-center clinical trials are currently underway to further confirm the safety and efficacy of RESIN [187].

Another Mg-Zn-Ga alloy was used in a preclinical study in goats (Fig. 12f) [189]. Mg-Zn-Ga nails were implanted trans-epiphysally into the proximal tibial physis of juvenile sheep until skeletal maturity to investigate the influence of biodegradable metal on epiphyseal growth (Fig. 12g). After long-term follow-up of up to 3 years, there was no evidence of growth impairment (Fig. 12h). Metal is stronger than polymers and the recent discovery of the Zn-Li alloy by our group opens new doors for further potential applications in children [190].

Detailed materials used, treatment methods and clinical results are shown in Table 4 and Fig. 12.

**Table 4:** Relevant studies with different types of intramedullary nails made of resorbable materials for use in the pediatric population.

Year/Journal	Study model	Implant type and material	Methodology and research questions	Key findings
2013, Scand J Surg [184]	Preliminary technical report of clinical study	• PLGA (Activa IM nail™)	• Forearm shaft fractures. • Combination of IM-nails and casting. • Follow-up after at least 12 months.	• One re-fracture due to high-energy trauma. • Good fracture union and acceptable alignment in follow-up.
2018, Biomaterials [185]	Clinical study	• PLGA (Activa IM nail™) • Ti (TEN®)	• Forearm shaft fractures in children. • Evaluation of bone healing, pain and ROM of forearm, elbow and wrist. • Follow-up after 2 years.	• No difference between groups in ROM and residual angulation. • 2 implant failures among adolescents in PLGA group. • 2 re-injuries after bone union in adolescents of PLGA group. • Implant stability assessment among older children is needed.
2021, J Clin Med [186]	Retrospective controlled study	• PLGA (Activa IM nail™) • Ti (TEN®)	• Forearm shaft fractures in children. • Evaluation of degradation and bone healing. • Follow-up until at least 4 years.	• No clinically significant difference in recovery between groups. • 57.7 % of PLGA nails completely degraded after mean 6.8 years. • 2 implant failures 3 months post PLGA nails treatment.
2022, Children [159]	Prospective multicenter study	• PLGA (Activa IM nail™)	• Diaphyseal forearm fractures in children. • 76 patients from 9 clinical pilot are analyzed • Follow-up for one year.	• Equal performance of PLGA nails in comparison to TEN. • The use of Activa IM-Nails seems to be equal to the standard TEN procedure compared to the literature.
2022, JCO [187]	Clinical pilot study	• PLGA (Activa IM nail™)	• Forearm shaft fractures in children. • Evaluation of soft tissue complications by MRI. • Follow-up for 78–203 days.	• 33.3 % showed transient signal pathology in a tendon. • Edema around superficial radial nerve in 86.7 %. • Caution with surgical preparation of soft tissue cleavage.
2024, Children [188]	Retrospective controlled study	• PLGA (Activa IM nail™) • Ti (TEN®)	• Pediatric forearm fractures. • Comparation of patient demographics and complications between PLGA implants and TEN.	• PLGA group (7 %) have lower complication than TEN (20 %). • The advantages of PLGA implants include no need for secondary surgery and associated cost savings and reduced complication rate and stress associated with anesthesia and surgery.
2023, Biomater Adv [189]	Animals experiments	• ZX10 (Mg-1Zn-0.3Ca, ESIN) • ZX00 (Mg-0.3Zn- 0.4Ca, ESIN)	• Transepiphyseal implantation of two Mg ESIN into the right tibia on four sheeps. • Investigated the influence on tibial physis. • Follow-up for over 3 years.	• Both ZX00 and ZX10 appear to be promising ESIN materials. • Transepiphyseal implantation of resorbable Mg-Zn-Ca implants without having an impact on growth mechanisms would extend the use of isolated intramedullary nails to the pediatric field.

## 5 Outlook for future

**Fig. 13:**
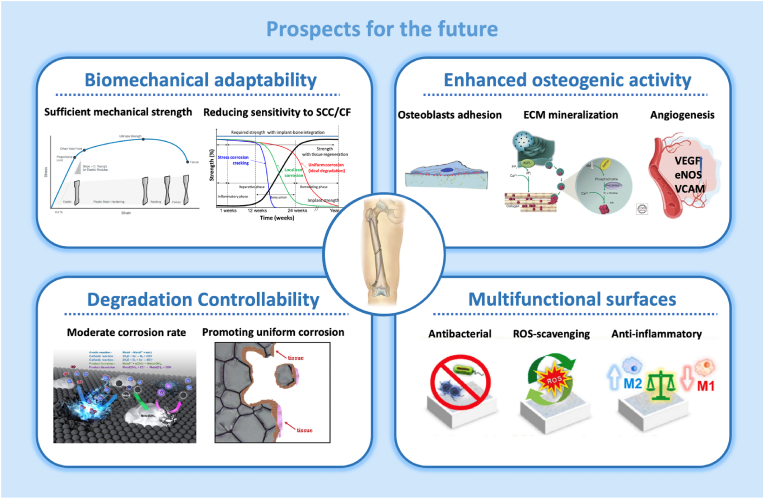
A schematic diagram summarizing the future prospects of utilizing the biodegradable alloy implants in pediatric orthopedics.

Biodegradable alloy implants show great potential in pediatric orthopedics, but still face several challenges. For Mg-based alloy implants, biomechanical adaptability is the first thing to consider. Typically, the coexistence of body fluid and external stress/strain will inevitably lead to the stress corrosion cracking (SCC) or corrosion fatigue (CF) of the implant [191], causing the rapid loss of mechanical integrity and implant failure. In recent decades, some modified technologies, such as heat treatment, surface modification, hot deformation, etc., have been adopted to ameliorate the SCC/CF sensitivity of the implants (as shown in Fig. 13) [[192], [193], [194], [195], [196], [197]]. In addition, the process of bone regeneration may suffer from the cathodic hydrogen evolution reaction, which causes the accumulation of hydrogen produced during in vivo degradation. The excessive H_2_ accumulation can lead to subcutaneous emphysema, delayed bone healing, and mechanical instability. To address this issue, alloying strategies focusing on refining microstructure and enhancing corrosion resistance have recently been adopted. For instance, the addition of rare earth elements (e.g., Y, Nd, Gd) and Zn forms intermetallic phases (e.g., Mg-Y, Mg-Zn-Ca) that act as cathodic inhibitors, reducing galvanic corrosion and stabilizing degradation rates [198]. The Mg-Y-RE-Zr alloy (e.g., WE43) demonstrates slower degradation due to the formation of a protective Y_2_O_3_-rich oxide layer, minimizing H_2_ release [199]. Similarly, trace calcium (Ca) incorporation promotes the formation of a Ca-P-rich surface layer in physiological environments, further decelerating corrosion [47]. Recent advances also explore ternary alloys (e.g., Mg-Zn-Mn) and heat treatments to homogenize grain boundaries, mitigating stress corrosion cracking (SCC) and localized pitting [200]. Computational modeling aids in optimizing alloy compositions for balanced mechanical integrity and degradation kinetics, as seen in Mg-Li-based systems [201], which exhibit improved ductility and uniform degradation. Preliminary results indicate that combining theoretical calculations with experimental validation of alloying can more effectively address issues such as the excessively rapid degradation rate and hydrogen evolution in Mg-based alloys. In addition, surface coatings are pivotal in modulating Mg alloy degradation. Microarc oxidation (MAO) creates a dense ceramic oxide layer (e.g., MgO/Mg_3_(PO_4_)_2_) that delays initial corrosion and H_2_ evolution [202]. Hybrid coatings, such as PLGA infused with hydroxyapatite (HA), combine barrier protection with bioactivity, enhancing osteointegration while neutralizing acidic by-products [203]. Recent breakthroughs include “smart' pH-responsive coatings (e.g., chitosan/gelatin) that release corrosion inhibitors (e.g., vanadate) in acidic microenvironments [204], counteracting rapid degradation during inflammation. Additionally, atomic layer deposition (ALD) of ultrathin Al_2_O_3_ or ZnO films provides nanoscale protection without compromising mechanical flexibility [205]. These multifunctional coatings not only mitigate gas generation but also synergize with alloy design to tailor degradation profiles for pediatric applications. More importantly, improved corrosion resistance and uniform corrosion mode are also desired for osteoimplants, which can provide sufficient Mg^2+^ release and mechanical support during bone repair. With regard to Zn-based osteoimplants, the main drawback limiting their clinical application is low cytocompatibility with osteoblasts. Methods to improve osteoblast adhesion, ECM mineralization, and angiogenesis are needed to enhance osteogenic activity. For Mg, Zn, or other recently proposed promising degradable metals, such as molybdenum and tungsten, further research is neededto develop novel architectures, coatings, and modification methods that will improve the biological effects and degradation properties of the implant.

## 6 Conclusion

This review demonstrated the characteristics of children's skeletal development, injury types and corresponding pediatric osteoinplants, analyzed and compared the characteristics of absorbable polymer materials and biodegradable metals. We also specifically listed the three frequrently used inplants (Pins, Screws and Intramedullary Nails) in pediatric orthopedics, exhibited and discussed their application in skeleton immature children. The detailed comparison and future outlook can provide a good reference for orthopedic surgeons in osteoinplant selection and material experts in the future development of biodegradable materials for children. Through the continuous updating and improvement of materials and the gradual clinical applications, it won't be a dream to extensively expand application of degradable materials in pediatric orthopedics in the future, which may shift from absorbable polymers to biodegradable metals.

## CRediT authorship contribution statement

**Yunan Lu:** Writing – review & editing, Writing – original draft, Visualization, Methodology, Funding acquisition, Conceptualization. **Ting Zhang:** Software, Methodology, Formal analysis. **Kai Chen:** Writing – review & editing, Writing – original draft, Methodology, Investigation, Formal analysis. **Federico Canavese:** Writing – review & editing, Investigation, Formal analysis. **Chenyang Huang:** Software, Investigation, Conceptualization. **Hongtao Yang:** Investigation, Conceptualization. **Jiahui Shi:** Software, Methodology. **Wubing He:** Visualization, Validation, Methodology. **Yufeng Zheng:** Writing – review & editing, Visualization, Investigation, Conceptualization. **Shunyou Chen:** Writing – review & editing, Resources, Project administration, Investigation, Conceptualization.

## Ethics approval and consent to participate

Not applicable.

## Declaration of competing interest

The authors declare the following financial interests/personal relationships which may be considered as potential competing interests: Yufeng Zheng is an editor-in-chief for Bioactive Materials and was not involved in the editorial review or the decision to publish this article. Other authorsdeclare that they have no known competing financial interests or personal relationships that could have appeared to influence the work reported in this paper.
